# *Haematococcus lacustris* genome assembly and annotation reveal diploid genetic traits and stress-induced gene expression patterns

**DOI:** 10.1016/j.algal.2024.103567

**Published:** 2024-06

**Authors:** Luca Marcolungo, Francesco Bellamoli, Michela Cecchin, Giulia Lopatriello, Marzia Rossato, Emanuela Cosentino, Stephane Rombauts, Massimo Delledonne, Matteo Ballottari

**Affiliations:** a Dipartimento di Biotecnologie, Università di Verona, Strada Le Grazie 15, 37134 Verona, Italy; b Bioinformatics and Evolutionary Genomics, University of Ghent, Technologiepark 927, B-9052 Gent, Belgium

**Keywords:** *Haematococcus*, Astaxanthin, Ploidy, Genome assembly, Microalgae, Next-generation sequencing

## Abstract

The green alga *Haematococcus lacustris* (formerly *Haematococcus pluvialis*) is a primary source of astaxanthin, a ketocarotenoid with high antioxidant activity and several industrial applications. Here, the *Haematococcus lacustris* highly repetitive genome was reconstructed by exploiting next-generation sequencing integrated with Hi-C scaffolding, obtaining a 151 Mb genome assembly in 32 scaffolds at a near-chromosome level with high continuity. Surprisingly, the distribution of the single-nucleotide-polymorphisms identified demonstrates a diploid configuration for the *Haematococcus* genome, further validated by Sanger sequencing of heterozygous regions. Functional annotation and RNA-seq data enabled the identification of 13,946 nuclear genes, with >5000 genes not previously identified in this species, providing insights into the molecular basis for metabolic rear-rangement in stressing conditions such as high light and/or nitrogen starvation, where astaxanthin biosynthesis is triggered. These data constitute a rich genetic resource for biotechnological manipulation of *Haematococcus lacustris* highlighting potential targets to improve astaxanthin and carotenoid productivity.

## 1 Introduction

Microalgae are unicellular photosynthetic organisms that can be cultivated in artificial cultivation systems to harvest light energy and use it to fix CO_2_ into biomass and bioproducts of interest. One of the most widely considered bioproducts that can be produced in microalgae nowadays is astaxanthin [1,2]. Astaxanthin is a ketocarotenoid with several industrial applications due to its red pigmentation and extraordinary antioxidant power [3–5]. Astaxanthin is used in the food and feed industry, nutraceutical products, and cosmetics [1,6]. The United States Food and Drug Administration (USFDA) defined natural astaxanthin as GRAS (Generally recognized as safe), and the European Food Safety Authority (EFSA) recommended the daily intake of 0.034 mg/Kg of astaxanthin for humans, with the consumption of this molecule being reported to improve human health thanks to its anti-inflammatory and antioxidant properties [7]. *Haematococcus lacustris* (formerly called *Haematococcus pluvialis*), a green unicellular alga, is the primary species used at the industrial level to produce natural astaxanthin [8,9]. In particular, astaxanthin biosynthesis occurs in *H. lacustris* when cells are exposed to stress conditions, such as high light, high or low temperatures, nutrient starvation, and/or high salinity, among others [9–17]. In these conditions, carotenoid biosynthesis is triggered, leading to the export of beta-carotene from the plastid to the cytosol, where the enzyme beta-carotene ketolase (BKT) catalyzes its ketolation, producing astaxanthin, which can be accumulated up to 4–5 % of cell dry weight [18,19]. Astaxanthin accumulation increases photoprotection in *H. lacustris*, mainly protecting DNA from oxidation and UV damage [20–22]. The complex mechanism at the base of stress-dependent astaxanthin accumulation in *H. lacustris* has not yet been fully elucidated: it is triggered by ROS (Reactive Oxygen Species) being produced due to stress exposure [23], but the different components of signaling pathways and the transporters of carotenoids from the plastid to the cytoplasm, where the ketolation reaction takes place, are essentially unknown.

Despite the possibility of producing “natural” astaxanthin by microalgae cultivation, most of the astaxanthin in the market is obtained by chemical synthesis from petrochemical precursors [24]: while the spectral properties of natural and synthetic astaxanthin are similar, the antioxidant power of the synthetic one is far lower compared to natural astaxanthin [25]. This difference is caused by the production of a mixture of byproduct stereoisomers, inherent to chemical synthesis, with different chemical properties compared to the stereoisomers mainly found in the astaxanthin molecules produced by microalgae [25]. For this reason, synthetic astaxanthin cannot be used for human consumption [26]. Astaxanthin production by microalgae cultivation is, however, a costly process, with the biomass production yield extremely low upon *H. lacustris* cultivation. Moreover, controlling the cultivation conditions to ensure proper biomass accumulation before inducing astaxanthin production leads to high OPEX (operating expenses) and CAPEX (capital expenditures) costs, and extracting pigments from stressed cells requires harsh methods [24,27]. Upon stress induction, *H. lacustris* cells shift to a cyst cellular phase where the cell dimension increases, and the cell wall gets extremely tough. Indeed, supercritical CO__2__ is the standard method used to extract astaxanthin from microalgal cells efficiently [27,28]. Developing new *H. lacustris* strains to boost astaxanthin productivity require advanced biotechnological solutions for strain manipulation, such as genome editing, which can be optimized only if high-quality genome assemblies are available. In the case of *H. lacustris*, only incomplete and highly fragmented genomes are available in the literature [29,30]. In 2019, a first genome draft was released for the strain SAG 192.80 based on Illumina sequencing, resulting in a genome size of 669 Mb, an N50 of 8.2 kb (increased to 288.6 kb for the scaffold sequences), and 18,545 predicted genes [29]. Later on, a second *H. lacustris* genome draft was made available in 2020 for the NIES-144 strain based again on Illumina pair-end sequencing: in this case, a genome size of 172 Mb in 9693 scaffolds with an N50 of 38.9 kb and 13,309 predicted genes were reported [30]. In parallel with the writing of this work, the genome draft of SAG 192.80 has been updated with a new genome assembly based on Pac-Bio long reads and Hi-C sequencing, with authors identifying 32 putative haplotypic chromosomes with an N50 scaffold of 942.6 kb [31] but with almost double genome size (309 vs. 172 Mb) compared to the NIES-144 strain genome assembly [30]. Besides the nuclear genome, extraordinarily large chloroplast (~1.35 Mb) and mitochondrial (~126 kb) genomes were recently released by independent reports, being the largest chloroplast and mitochondrial genomes found in *Chlorophyta* up to now [32–34]. The absence of complete *Haematococcus* genomes with high contiguity hamper the onset of advanced biotechnological tools as CRISPR/CAS genome editing methods, which require complete and fully sequenced genome assembly to identify possible off-target sites [35]. Moreover, accurate genome annotation and identification of the key genes at the base of the stress response in *H. lacustris* is required to design possible metabolic engineering strategies to improve astaxanthin production in microalgae.

In this work, *Haematococcus* strain K-0084 was reconstructed at a near-chromosome level for the nuclear genome with complete chloroplast and mitochondrial genomes to unravel the genetic information underlying *Haematococcus* features. The high-quality genome assembly retrieved demonstrates a diploid organization of the genetic information in this photosynthetic organism and allows for a detailed analysis of gene expression patterns upon exposure to astaxanthin-inducing stresses such as high light or nitrogen starvation.

## 2 Materials and methods

### 2.1 Haematococcus lacustris cultivation

*Haematococcus lacustris* strain K-0084 was obtained from the Scandinavian Culture Collection of Algae & Protozoa. Liquid cultures were grown photoautotrophically at 40 μmol photons m^−2^ s^−1^ on BG-11 medium at 22 °C in flasks [11]. Cells at the exponential phase (approximately 5 × 10^5^ cells ml^−1^) were then exposed for three days to four different growth conditions, being low light (40 μmol photons m^−2^ s^−1^) or high light (800 μmol photons m^−2^ s^−1^) in nitrogen-replete medium (samples named respectively LL and HL) or in nitrogen starvation (samples named LL-N or HL-N for cells grown in nitrogen starvation respectively at 40 and 800 μmol photons m^−2^ s^−1^). 17.65 mM of sodium nitrate was used as a nitrogen source in the nitrogen replete condition (LL and HL), while in the case of nitrogen starvation (LL-N and HL-N samples), no nitrate was added to the growth medium. In the case of LL-N and HL-N samples, before the exposure to nitrogen starvation, cells were harvested by centrifugation and washed three times with BG-11 medium prepared without nitrate. Experiments were done on a rotative shaker (150 rpm) to prevent cell sedimentation and induce gas exchange and repeated at least five times independently with three biological replicates for each sample.

### 2.2 Pigments analysis

Pigments were extracted from intact cells using DMSO [36] as described in [11]. The chlorophyll to carotenoid ratio and chlorophyll *a*/b ratio were estimated from the absorption spectra of pigment extracts as described in [37]. Carotenoid content was analyzed by HPLC [37].

### 2.3 ONT sequencing

Nuclei were isolated from 4.3 × 10^8^ *H. lacustris* cells in MEB buffer [38] and the nuclear DNA was extracted using the Qiagen Genomic Tip-100 (Qiagen, Hilden, Germany). After DNA quantification and quality control as above, the nuclear DNA was fragmented to ~20 kb using a g-TUBE (Covaris, Brighton, UK) and treated with short read eliminator (Circulomics, Pacific Biosciences) to remove short fragments [39]. A 4-μg aliquot of DNA was end-repaired and dA-tailed using the Next End Repair/dA-tailing module (New England Biolabs, Ipswich, MA, USA), and ONT libraries were prepared using the ligation protocol (SQK-LSK109) according to the manufacturer’s instructions (ONT, Oxford, UK). Approximately 15 fmol of the library was loaded into a MinION flow cell (FLO-MIN106_R9.4.1) and loading was repeated on the same flow cell after nuclease flushing (NFL_9076_v109). The sequencing run lasted ~48 h generating 2 million reads corresponding to12Gbp (Supplementary Table 1).

### 2.4 Illumina sequencing

Whole-genome Illumina DNA-Seq libraries were prepared using the KAPA Hyper Prep Kit (Kapa Biosystems, Wilmington, MA, USA) and a PCR-free protocol, starting from the same DNA utilized for ONT sequencing. Nuclear DNA was sheared using an M220 ultra-sonicator (Covaris), adjusting the treatment time to obtain ~350-bp fragments. The size of the resulting libraries was assessed by capillary electrophoresis on a Bioanalyzer High Sensitivity DNA chip. Libraries were quantified by qPCR using a standard curve and were sequenced on an Illumina NovaSeq6000 to generate 119 million 150 paired-end reads (36 Gbp, Supplementary Table 1).

### 2.5 PacBio sequencing

DNA was extracted from 500 ml of 4.3 × 10^8^ *H. lacustris* cells using the CTAB (Cetyl trimethyl ammonium bromide) extraction buffer. Extracted DNA was treated with 200 μg/ml RNAase A at 37 °C for 20 min and subsequently purified with 1,8× AMpureXP beads (Agencourt). After DNA quantification and quality control as above, two independent single-molecule real-time (SMRT) bell libraries according to the manufacturer’s protocol (Pacific Biosciences; 20-kb template preparation using BluePippin (SageScience) size selection system with a 15-kb cut-off) were prepared. Sequencing was performed on a PacBio RS-II platform (Pacific Biosciences, CA, USA) using PacBio P6-C4 chemistry generating 2.8 million reads corresponding to 21.8 Gbp (Supplementary Table 1).

### 2.6 Hi-C data generation

*H. lacustris* biomass was fixed in 1 % fresh formaldehyde for 20 min followed by 1.25 mM glycine incubation. Nuclei were isolated in NIBTM buffer [40], and Hi-C libraries were prepared using the Proximo Hi-C Plant kit v1.5 (Phase Genomics, Seattle, WA, USA) and restriction enzyme *Sau*3AI. The integrity and size distribution of the Hi-C library were assessed using a 2200 TapeStation (Agilent Technologies, Santa Clara, CA, USA). The number of Hi-C library sequenced fragments was 302,601,482 which were sequenced on the NovaSeq sequencing platform using the 150 paired ends protocol.

### 2.7 Genome assembly

ONT raw reads were assembled using Flye v2.5 with default parameters [41] obtaining the first draft of genome assembly. The draft contig assembly underwent base-level refinement of residual errors using a combination of long and short reads. Briefly, ONT reads were aligned on the ONT draft assembly using minimap2 v2.17 with the -x map-ont parameter [42]. Racon v1.4.3 [43] was used for the initial polishing of long reads, followed by a second round using medaka v1.0.3 (https://github.com/nanoporetech/medaka) and two rounds of sequence refinement using short reads in Pilon v1.23 [44]. Genome completeness was assessed with BUSCO v4.0.6 [45] using chlorophyta_odb10 as a reference database. The purging and scaffolding of the polished ONT assembly using Hi-C data was carried out by Phase Genomics using the Proximo Genome Scaffolding Platform. Finally, Illumina reads were aligned on the scaffolded genome assembly using BWA mem v0.7.17-r1188 [46] and duplicates were removed with Picard MarkDuplicates (http://broadinstitute.github.io/picard/). Variants were identified using freebayes v1.3.6 [47] using -m 20 -q 20 –min-coverage 10 parameters. Telomeric motifs were analyzed at the scaffold ends as previously reported [48] by using a telomeric-identifier toolkit (https://github.com/tolkit/telomeric-identifier) and telomeric motif associated with *Chlamydomonales* or previously identified in *C. reinhardtii* genome [49]. The assembled scaffolds of *H. lacustris* genome was aligned to the assembled scaffolds of previously released *H. lacustris* genome by Bian and coworkers [31] using Mashmap2 [50]. The result was then visualized as a dot plot using D-GENIES [51].

### 2.8 Organelle genome assembly

The organelle genomes were assembled using the Organelle_PBA pipeline [52] using the *H. lacustris* strain UTEX 2505 chloroplast and mitochondrial genomes as reference (NCBI accession number MG677935.1 and MK878592.1, respectively). The sequences were then polished using long and short read data. Briefly, PacBio reads were aligned to the organelle genome assemblies using pbalign v0.2.0.138342 before polishing with ConsensusCore v0.8.8 using the quiver algorithm (https://github.com/PacificBiosciences/pbbioconda). Illumina reads were then aligned to the polished long-read assembly using BWA mem v0.7.17-r1188 [46], and pilon v1.23 [44] was used for base-level refinement. Finally, the alignment between the ONT assembly and the organelle genomes was performed using Blastn (v2.9.0) [53]. Those ONT contigs aligning to the organelle genome with a similarity of at least 99 % were manually removed.

### 2.9 Analysis of the ploidy of the genome assembly

The ploidy level of the *H. lacustris* genome assembly was investigated by evaluating the distribution of Illumina reads in the alignment using nQuire [54] and ploidyNGS [55]: both methods assume that alleles (SNPs frequency) occur at different ratios for different ploidy levels: 0.5/0.5 in diploids, 0.33/0.67 in triploids, and a mixture of 0.25/0.75 and 0.5/0.5 in tetraploids. nQuire built three Gaussian Mixture Models (or GMM), one for each level of ploidy (diploid, triploid, and tetraploid), and calculated the logarithmic distance between each model and the data distribution. PloidyNGS analyzes the distribution of alleles (or SNPs) distribution [55]. A second approach to validate the diploidy of *H. lacustris* was based on Sanger sequencing and propagation of SNPs in cell progeny: 8 different SNPs in heterozygosis were selected, amplified by PCR and sequenced by Sanger sequencing in isolated colonies. *H. lacustris* cells were thus propagated in different plates, single colonies were picked up and grown in 20 ml flasks, and the genomic DNA was extracted. The different DNA extracts were then amplified in specific regions where heterozygous SNPs had been identified in the final genome assembly.

### 2.10 RNA extraction and RNA-seq analysis

The samples for RNA extraction were harvested after three days of growth in the four conditions described in Section 2.1 (LL, LL-N, HL and HL-N). To ensure minimal variation, all samples were collected for RNA extraction and processed on the same day. RNA was extracted from 500 ml of *H. lacustris* liquid culture with a density of 5 × 10^5^ cell/ml using the TRIzol SIGMA-ALDRICH protocol, then RNA samples were further purified with the SIGMA Spectrum Plant Total RNA kit including a DNAse treatment step. RNA quality and quantity were determined using a NanoDrop 2000 spectrophotometer (Thermo Fisher Scientific) and a Bioanalyzer Chip RNA 7500 series II (Agilent Technologies), respectively. Directional RNA-Seq libraries were prepared from 1 μg total RNA using the TruSeq RNA Sample Prep Kit v2 (Illumina) after capturing poly-adenylated transcripts. Libraries were checked for quality using a High Sensitivity DNA Kit on a 2200 TapeStation device, quantified by qPCR using primers annealing to the adapter sequences, and sequenced in 75 paired-end reads on a NextSeq500 (Illumina), generating 21.9 M fragments per sample on average.

### 2.11 Nuclear gene annotation

Repetitive elements were identified using RepeatModeler v 2.0.1 [56] with LTR structural search pipeline. After repetitive elements were annotated, the *Haematococcus* genome was soft-masked using the RepeatMasker v 4.1.1 program [57]. Sequenced RNA-seq data was exploited for gene prediction. Firstly, Illumina RNA-seq data were filtered to remove low quality reads and Illumina sequencing adapter. Specifically, low-quality reads (defined as those reads with >50 bp lower than phred quality 7 or containing >10 % of undetermined bases) were discarded using sickle v1.33 (https://github.com/najoshi/sickle), and Illumina sequencing adapter was removed using scythe v0.994 (https://github.com/vsbuffalo/scythe). Filtered reads were aligned to the genome using HISAT2 v 2.2.1 aligner with max_intron_length equal to 50 kbp [58]. Available PacBio Isoform sequencing data (SRR6816386) [29] were aligned against the genome with GMAP software (version 2017-11-15) with intron length equal to 50kpb, with -n parameters set to 0 and with the PASA pipeline v2.3.3. *Arabidopsis thaliana* TAIR10, *Chlamydomonas reinhardtii* v5.6, and *Volvox carterii* v2.1 proteins were aligned using GenomeThreader v 1.7.1 [59]. Subsequently, the generated alignments of protein and RNA-seq (both Illumina and PacBio) data were converted into hints, which were provided to Augustus predictor as external evidence. BUSCO v5 [60] was used to train the model specific for the *Haematococcus* genome using *Chlorophyta* BUSCO genes. Subsequently, Augustus 3.3.3 [61] was run using the trained-specific model with so-called hints supported by protein and RNA data. Functions of annotated protein-coding genes were defined with a custom script that integrates homology information from BLASTp matches [53], orthology inference together, and annotation of function domains. In brief, predicted proteins were aligned with BLAST v 2.2.28+ against TAIR10 and *Chlamydomonas* annotations to infer a function. Orthology inference of predicted proteins was performed using Orthofinder v 2.4.0 software [62] together with proteins annotated in *Arabidopsis thaliana* TAIR10, *Chlamydomonas reinhardtii* v5.6, *Volvox carteri* v2.1, *Chlorella variabilis* NC64A v1.0, *Ostreococcus tauri* v3.0 and *Dunaliella salina* v1.0. In addition, protein domains and motifs were searched using the InterProScan 5.46–81.0 program using default databases [63]. Annotated sequences were analyzed by the KAAS (KEGG Automatic Annotation Server) platform to obtain KO annotation [64–66]. Transcripts differently expressed with KO annotation were visualized by the KEGG Mapper platform, while the remaining transcripts functionally annotated were manually inspected by retrieving the function of the closest homolog gene. Whenever a gene of interest was missing in the list of genes annotated by KEGG Mapper, we turned to homologs from high-quality genomes of closely related species for a hmmer/BLAST search against the translated transcriptome of *H. lacustris*. If identified, the closest homolog’s translated transcript was then scrutinized using two approaches: first, through a BLASTp search against the NCBI non-redundant (nr) database to establish its homology with genes of known function in other species that possessed the function we were investigating, and second, by employing domain analysis tools such as InterPro and Pfam to verify the congruence of the protein domains. Moreover, the presence of apparently missing genes in the *H. lacustris* genome was further investigated in the RNA-seq data utilizing HISAT2 set to a higher-than-standard mismatch tolerance, aiming to align reads with known sequences of homologs from closely related species. Anyway, none of the gene not found in the genome assembly could be find in the RNA-seq data.

### 2.12 Phylogenetic analysis

Phylogenetic analysis was performed by BUSCO analysis as previously reported [67]. In particular, BUSCO was employed to identify orthologous genes in the analyzed genome assemblies, and among the identified genes, 114 single-copy genes shared between all the assemblies were used for the phylogenetic analysis by protein alignment and phylogenetic tree construction. For each protein, a multiple alignment was performed with MUSCLE 3.8.31 [68], and then the alignments were concatenated. The tree was built using the web application Phylogeny.fr running PhyMl and TreeDyn using default parameter for the construction and the visualization, respectively [69].

### 2.13 Organelle genome annotation

In organelle genomes, protein-coding genes were predicted using PROKKA v1.14.6 [70] using plant chloroplast and mitochondrial genes deposited in the NCBI RefSeq protein database. In parallel, genes were annotated using Geseq [71] using *C. reinharditii* and *H. lacustris* annotation. In the chloroplast genome, [72] and [33] annotations were transferred using liftoff v1.6.3 [73], and the procedure was used for the mitochondrial genome to transfer [34] annotations. Subsequently, the gene models with the most correct ORF were chosen among PROKKA, Geseq, and liftoff predictions. In addition, manual curation was performed on 49 gene models for the chloroplast and 8 for the mitochondria. Internally to the Geseq pipeline, tRNA and rRNA genes were predicted using tRNA-scanSE [74] and BLAT [75] respectively. Genome maps for both chloroplast and mitochondrion were generated with OGDRAW (version 1.3.1) [76].

### 2.14 Identification of simple sequence repeat

Simple sequence repeats were identified using TandemRepeatFinder v4.0.6 using 2 7 7 80 10 502,000 -d—h parameters in *Chlamydomonas reinhardtii* v5.6, Volvox carterii v2.1, and Haematoccus lacustris CDS sequences. The resulting tandem repetitive elements were filtered based on the length of the consensus sequence to retain only di-, tri-, tetra-, or pentanucleotide motifs.

### 2.15 Differential gene expression and enrichment analysis

Pseudoalignments to estimate transcript counts for Illumina RNA-seq data were performed with Salmon (version 1.9.0) [77]. Counts were summarized at the gene level with tximport (version 1.26.0), while differential gene expression analysis was conducted with DESeq2 (version 1.38.1) [78], setting a *p*-value threshold of 0.05 and a log2 fold threshold of 0.58 (equivalent to a fold change of 1.5). The adaptive shrinkage (ashr) method was incorporated within DESeq2 as a shrinkage estimator for more accurate effect size estimation [79]. Detailed annotation of Gene Ontology (GO) terms was obtained by using Pannzer2 [80]. Differential gene expression analysis was performed both for low light vs. high light (both with and without nitrogen starvation, individually) and for repleted nitrogen vs. nitrogen starvation (both in low light and high light conditions, individually). Enrichment analysis was performed with clusterprofile R package (version 4.6.0) with a p-value cutoff of 0.05 [81]. and enrich plot (version 1.18.3) [82].

### 2.16 Subcellular localization prediction

Subcellular localization prediction was performed by using the PredAlgo tool as previously described [83]. Briefly, PredAlgo is based on a neural network that has been trained using carefully curated sets of *C. reinhardtii* proteins to predict intracellular localization in the mitochondrion, the chloroplast, and the secretory pathway.

## 3 Results

### 3.1 Development of a high-quality reference genome sequence of Haematococcus lacustris

**Fig. 1:**
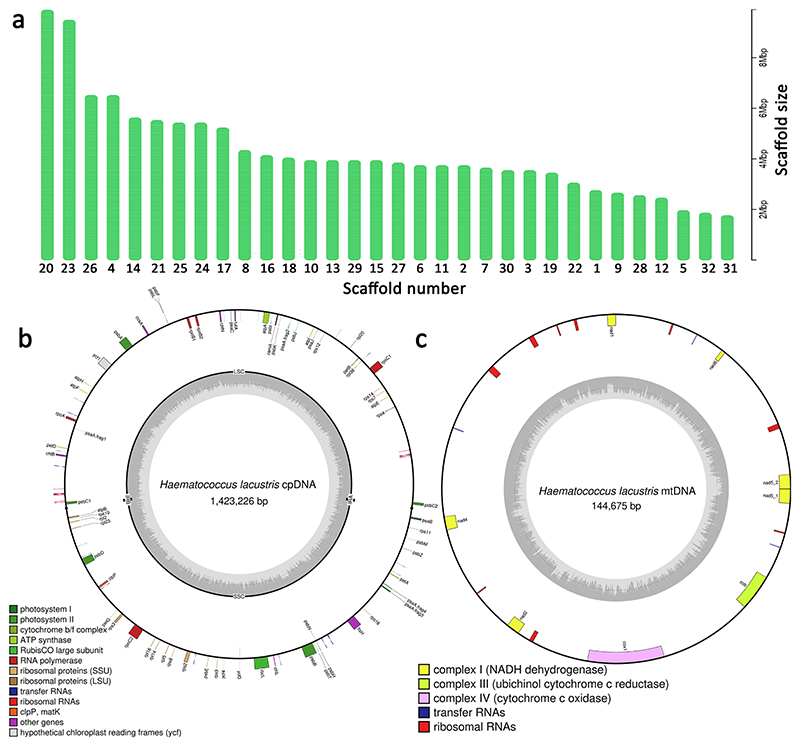
Assembled *Haematococcus lacustris* nuclear genome. (a) *Haematococcus lacustris* nuclear genome was assembled in 32 pseudo-molecules based on the integration of next-generation sequencing (NGS) and Hi-C scaffolding as described in the main text. Unplaced contigs are reported to represent 0.81 % of the *Haematococcus lacustris* genome. (b) Chloroplast and (c) mitochondrial genomes of *Haematococcus lacustris* assembled based on NGS sequencing data. The location of putative genes and their direction of transcription are indicated: genes located outside the circle are transcribed in a clockwise direction, whereas those positioned inside the circle follow a counterclockwise transcription path. The inner circle, shaded in grey, depicts the GC content, with a darker grey line at the center marking the 50 % GC content threshold.

To investigate the genetic basis underlying the phenotype of *Haematococcus lacustris*, we sequenced, assembled, and functionally annotated its nuclear genome. Genome assembly was obtained by integrating different genomic approaches displaying complementary features, i.e., Oxford Nanopore Technologies (ONT) for long-reads, Illumina for accurate short-reads, and Hi-C scaffolding for reaching high continuity of the assembly (Supplementary Fig. 1). High coverage (~40 ×) raw ONT reads (Supplementary Table 1) were assembled into a draft genome assembly of 272 Mb, consisting of 4645 contigs and N50 of 250 kb. Illumina short reads were used to polish the ONT-based genome assembly which was then evaluated by BUSCO genome analysis [45,67]. BUSCO analysis was performed on a benchmark of 1519 genes (chlorophyta_odb10 BUSCO database) putatively found in a single copy to assess the genome assembly and annotation completeness. Most of the BUSCO genes were identified in the *H. lacustris* draft genome (97 %) but surprisingly >74 % of these genes were identified as duplicated. Based on these results, an alternative genome assembly was obtained by purging contigs, assuming a possible diploidy feature of the *H. lacustris*. The purged genome assembly was characterized by a 150 Mb genome size represented by 1799 contigs with an N50 of 230 kb and an average contig size of 83 kb: in this case, BUSCO analysis resulted in genome completeness of 92 % with 10.6 % of BUSCO gene duplication. Finally, Hi-C scaffolding identified 32 scaffolds containing 91 % of the 151 Mb genome of *H. lacustris* (Fig. 1) with an N50 of 4 Mb (Table 1).

**Fig. 2:**
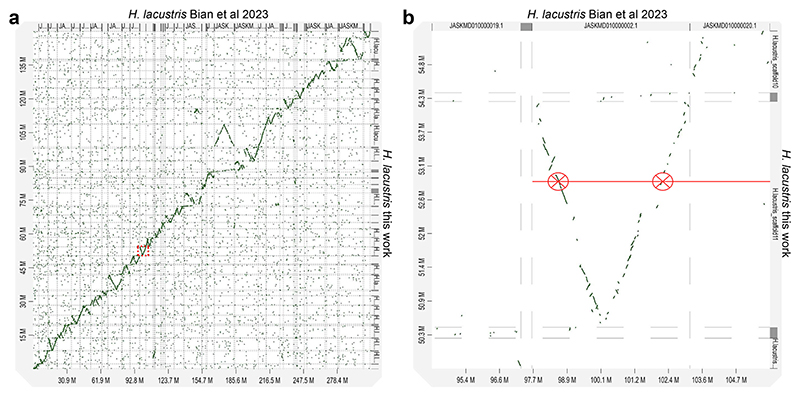
D-GENIES dot plot showing the alignment between previous and current *Haematococcus lacustris* genome assembly. a) Dot plot showing the complete alignment of *H. lacustris* genome assembly retrieved in Bian et al. 2023 and the genome assembly reported in this work (see Table 1 for the statistics of the two different genome assemblies). The X-axis represents the scaffolds reported in Bian et al. 2023 and Y-axis represents the scaffolds herein assembled. b) Focused view in the region indicated with a red rectangle in a) showing in red an example of double alignment between a sequence in one of the scaffolds herein assembled and one of the scaffolds reported by Bian and coworkers. (For interpretation of the references to color in this figure legend, the reader is referred to the web version of this article.)

The longest and the shortest scaffolds were, respectively, 9.9 Mb and 1.7 Mb. 10 of the remaining unplaced contigs were identified by subsequent analysis as part of the chloroplast and mitochondrial genomes, and they were therefore removed from the nuclear genome assembly: the remaining 839 unplaced contigs accounted only for <8.6 % of the genome size. Aligning the Illumina reads on the final scaffolded genome, a total of 2,314,504 heterozygous variants were found representing a degree of heterozygosity of 1.5 % (Supplementary Table 2). The same evaluation was performed for each assembled scaffold, resulting in an average degree of heterozygosity of 1.65 %, further suggesting a putative diploid feature of *H. lacustris* (Section 3.2). It is worth noting that the average Illumina coverage was relatively uniform across the different scaffolds (Supplementary Table 3). To further assess the quality of the assembled genome, putative telomeric motifs were searched at the ends of the scaffolds. As reported in Supplementary Table 4, several repeats of the AACCCT motif, previously reported as a telomeric repetitive motif in Chlamydomonadales (https://github.com/tolkit/a-telomeric-repeat-database), were found in all scaffolds herein assembled for *H. lacustris*. The assembled *H. lacustris* genome was thus aligned with the genome drafts previously released [31] to investigate their collinearity. As reported in Fig. 2a, the dot plot of the aligned genome showed a high collinearity relationship between the two-genome assemblies. However, most of the sequences present in the genome version herein assembled align twice against Bian et al.’s previous genome drafts (Fig. 2b). This finding is consistent with the proposed diploid feature of the *H. lacustris* genome herein adopted for genome assembly but not considered by Bian and coworkers in the previous genome draft.

### 3.2 Diploidy of the nuclear genome

The ploidy level of the *H. lacustris* genome was investigated further by two independent approaches following the single nucleotide variants present in the genome.

**Fig. 3:**
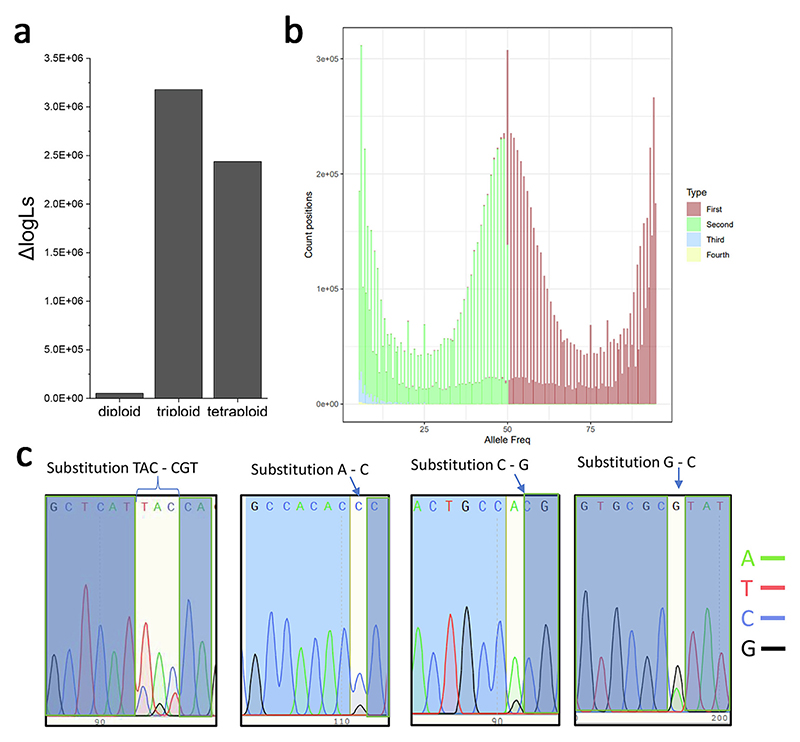
Analysis of ploidy in *Haematococcus lacustris*. Ploidy level estimation was carried out by evaluating the distribution of SNPs using two software packages: nQuire (a) and ploidyNGS (b). Both methods assume that alleles present at biallelic SNPs occur at different ratios for different ploidy levels: 0.5/0.5 in diploids, 0.33/0.67 in triploids, and a mixture of 0.25/0.75 and 0.5/0.5 in tetraploids. a) nQuire builds three Gaussian Mixture Models (or GMM), one for each level of ploidy (diploid, triploid, and tetraploid). A given ploidy level is supported by the smallest ΔlogLs value, describing the distance between each model and the «free» model built on the real distribution (a). b) PloidyNGS compares the SNPs distribution observed: the most frequent allele has a peak for monomorphic positions close to 95 %, and a peak close to 50 % for heterozygous positions, and the second most frequent allele has a peak close to 50 %, which represent heterozygous positions, and another one close to 5 % which represent sequencing errors, hence compatible with a diploid genome. The third and fourth most frequent alleles have a distribution peak of <5 %, suggesting being related to sequencing errors. c) Example of Sanger sequencing of regions with SNPs. DNA was extracted from isolated colonies, which, however, maintained the heterozygous features of SNPs.

In the first approach, Illumina reads were aligned on the genome assembly, and ploidy level estimation was carried out by evaluating the distribution of reads in the alignment assuming that alleles (SNPs frequency) occur at different ratios for different ploidy levels: 0.5/0.5 in diploids, 0.33/0.67 in triploids, and a mixture of 0.25/0.75 and 0.5/0.5 in tetraploids. Both software used, nQuire [54] and ploidyNGS [55], retrieved alleles distribution peak at 0.50, as expected for diploid organisms (Fig. 3a, b). These results are consistent with the diploid organization of the *H. lacustris* genome.

A second approach to validate the diploidy of *H. lacustris* was based on Sanger sequencing and propagation of SNPs in cell progeny (Fig. 3c). 8 different SNPs in heterozygosis were selected, amplified by PCR and sequenced by Sanger sequencing: in all cases, the sequencing results obtained demonstrated the presence of heterozygous SNPs. If the SNPs distribution was related to multiple *H. lacustris* genotypes in the culture used for sequencing, the heterozygous SNPs observed should be found as homozygous in isolated colonies. *H. lacustris* cells were thus propagated in different plates, single colonies were picked up and grown in 20 ml flasks, and the genomic DNA was extracted. The different DNA extracts were then amplified in specific regions where heterozygous SNPs had been identified in the final genome assembly. Among the tested 26 single colonies, the analyzed SNPs were identified as heterozygous in all cases (Fig. 3c), supporting the diploidy feature of the *H. lacustris* genome. It is important to note that even if the sexual reproduction of *H. lacustris* cannot be excluded in the conditions herein applied, the cultivation parameters previously reported to induce gametogenesis were not herein used [84].

### 3.3 Haematococcus lacustris nuclear genome annotation

Functional annotation of the newly assembled *H. lacustris* genome was performed by integrating ab initio gene prediction with RNA-seq data analysis (Supplementary Fig. 2). RNA-seq was performed on *H. lacustris* cells grown in four different conditions: low light (40 μmol m^−2^ s^−1^, LL), high light (800 μmol m^−2^ s^−1^, HL), low light in nitrogen starvation (LL–N), and high light in nitrogen starvation (HL–N) (Supplementary Fig. 3). It is important to note that in high light and/or in nitrogen starvation, *H. lacustris* accumulated astaxanthin, with HL-N being the growth conditions inducing the higher content of astaxanthin in the cells (Supplementary Table 5), as previously reported [11]. Short and Pac-Bio long reads [29] were considered for RNA-seq analysis and genome annotation (Supplementary Fig. 2).

**Fig. 4:**
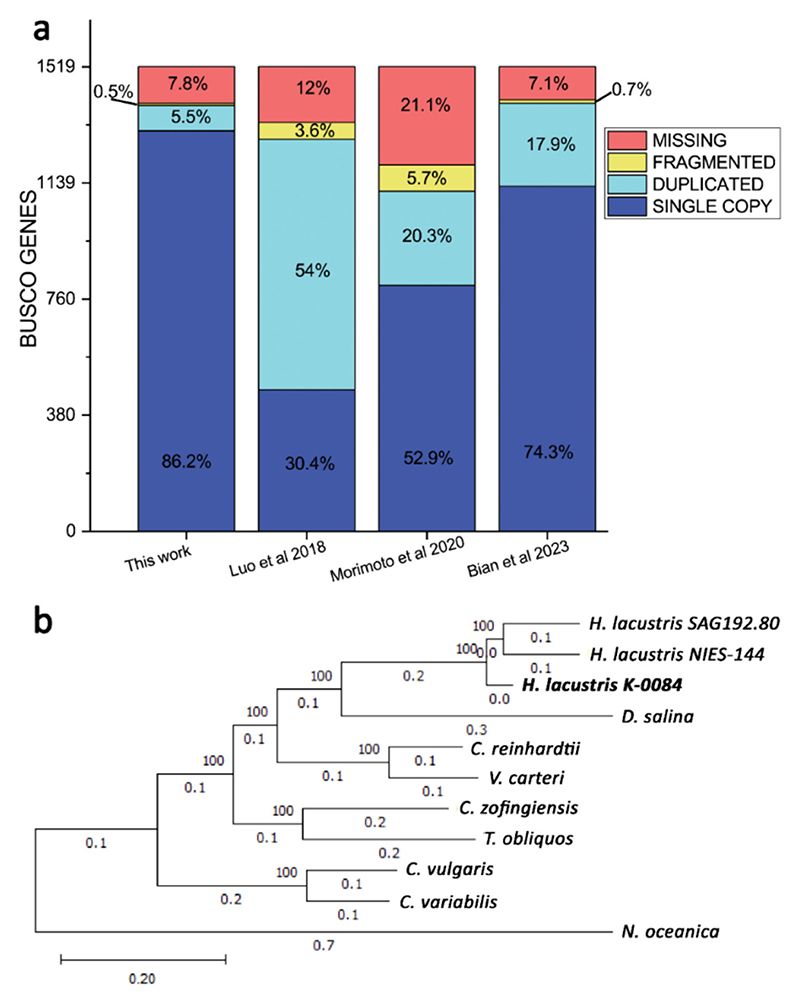
BUSCO analysis of *Haematococcus lacustris* genome and phylogenetic analysis. (a) BUSCO analysis of scaffolded *Haematococcus lacustris* genome assembled and annotated in this work in comparison to previous genome drafts available. Complete genes are reported as single-copy genes (blue) or duplicated genes (light blue). Fragmented genes identified are reported in yellow while missing genes are reported in red. (b) Phylogenetic tree of the *H. lacustris* K-0084 strain analyzed in this work compared to other microalgal strains with sequenced genomes. 114 single-copy genes shared with other species with an available genome were used for protein alignment and phylogenetic tree construction. The node labels represent the maximum likelihood bootstrap values of that node, ranging from 0 to 100, where 100 is the maximum confidence generated during the bootstrap iterations. The branch length, indicated with the number rounded at the first decimal digit below each branch, represents the average substitution rate of the analyzed 57,813 sites, namely the number of total amino acids in the 114 proteins employed for the tree construction. *H. lacustris* NIES-144 and *H. lacustris* SAG192.80 are the strains sequenced respectively in [30] and [29,31]. (For interpretation of the references to color in this figure legend, the reader is referred to the web version of this article.)

Genome annotation identified 13,946 genes (Supplementary dataset 1) with an average CDS length of 1397.12 bp and 10.37 exons per gene. BUSCO analysis was then performed on the final annotation, observing a genome completeness of 91.9 % and an annotation completeness of 90.9 %. Among these BUSCO genes, only 5.5 % were identified as duplicated, while 17.9 % of BUSCO genes were found duplicated in the most recent genome draft [31]. These numbers demonstrate an important increase in genome quality and completeness compared to previous *H. lacustris* genomes available (Fig. 4a). The total number of genes identified in this work, 13,946 genes, is similar to genes resulting from the annotation previously released by Morimoto and coworkers (13,309 genes) [30] but less than half of the genes reported by Bian and coworkers (30,505 genes) [31]. BlastP thus aligned the protein sequences identified by Morimoto and coworkers or Bian and coworkers with the protein sequences identified in this work: positive results were obtained for 78.8 % and 93.3 % of the protein sequences previously annotated respectively by Morimoto [30] and Bian [31] but aligning respectively only to 8895 or 8604 protein sequences identified in this work (Supplementary datasets 2 and 3). In several cases, multiple protein sequences identified in previous works clustered on a single longer protein sequence resulting from the genome annotation herein presented. These results suggest an increased number of genes identified with a reduced fragmentation compared to the previous annotation available. Functional genome annotation analysis reported Gene Ontology (GO) terms for 11,333 genes, representing 81.3 % of the predicted genes. The gene models predicted for *H. lacustris* were used to determine codon usage (Supplementary Table 7), which was similar to the codon usage observed in the case of *C. reinhardtii* [85,86] or *C. vulgaris* [87].

### 3.4 Phylogenetic analysis of Haematococcus lacustris

Functional annotation of the *H. lacustris* genome was then exploited to analyze the phylogenies of the strain (K-0084) herein investigated. In particular, 114 single-copy genes shared with other species with an available genome were used for protein alignment and phylogenetic tree construction. As reported in Fig. 4b, *H. lacustris* K-0084 is closely related to the NIES-144 and SAG192.80 *H. lacustris* strains previously sequenced [29–31]. Among other *Chlorophyta, Dunaliella salina* species was the closest species to *H. lacustris*.

### 3.5 Identification of genomic repeats

One of the critical points in the annotation pipeline was the repeats identification: as reported in Supplementary Table 6, 47.3 % of the genome appeared as composed of repeated sequences. To validate the approach adopted for repeats identification, the same analysis was performed in the case of the *C. reinhardtii* genome: in this case, 22 % of the genome appeared to be composed of repeated sequences, in line with previous findings Most interspersed repeats identified in the *H. lacustris* genome were short (< 2 kbp). Still, they were organized in tandem and formed complex patterns extending tens of kilobases in length (Supplementary Fig. 4). The longest stretch of composite repeats detected by manual inspection was 34 kbp (Supplementary Fig. 4).

The highly repetitive content of the *H. lacustris* genome led to further investigation into the presence of Simple Sequence Repeats (SSRs) in coding sequences. SSRs, or microsatellites, are DNA stretches consisting of short, tandemly repeated di-, tri-, tetra-or pentanucleotide motifs. Here, the presence of SSRs in coding sequences was investigated by SSR prediction. As reported in Table 2, only 5.2 % of *H. lacustris* genes were characterized by having SSRs in the coding sequence. A higher number of genes with SSR was identified in the *C. reinhardtii* or *Volvox carterii*, 18.2 % and 14.7 %, respectively (Table 2). As in the case of *C. reinhardtii* and *V. carteri*, most SSRs found in the *H. lacustris* coding sequence contain SSRs with block sizes of 3 corresponding to one translated codon. However, the average SSR size was larger in the case of *H. lacustris* compared to the other green algae investigated herein.

### 3.6 Chloroplast and mitochondrial genomes

Previous work reported a giant plastid genome size for *H. lacustris*, being up to 1.35 Mb [33,34]. The complete (circular) chloroplast genome of *H. lacustris* was reconstructed here with no gaps or ambiguous nucleotides, obtaining a length of 1.42 Mb (Fig. 1b). Structural annotation of genes encoded by the plastid genome revealed the presence of 65 protein-coding genes, 28 tRNA,13 rRNA, and 25 intron-encoded protein genes (Supplementary Table 8 and dataset 4). The number of genes identified in *H. lacustris* is consistent with previous findings in *C. reinhardtii* (Supplementary Table 8). The large size of the *H. lacustris* plastid genome is thus related to the presence of highly repeated DNA, as previously suggested [34]. Among the genes annotated, 28 encode for subunits of the complexes involved in the light phase of photosynthesis (PSI, PSII, cytochrome *b*6*f*, and ATP synthase), and one gene encodes for the large subunit of RUBISCO (*rcbL*). Among the other genes encoded by the chloroplast genome, *ycf1, ycf3*, and *ycf4*, but not *ycf2*, were herein identified, with the *ycf3* and *ycf4* being reported to be involved in PSI assembly [88]. Notably, several introns were identified in the plastid genes: introns in plastid genes were previously reported for other green algae, such as *C. reinhardtii* [89] or *Chlorella vulgaris* [87]. It is worth noting a peculiar feature found in intron regions of *atpA, ftsH, psaA, psbA, psbB, psbD, rbcL, rpoC1*, and *rpoC2*: here putative coding sequences for intron maturase, reverse transcriptase or deoxyuridine 5′-triphosphate nucleotide hydrolase enzymes were identified. The presence of intron maturase is a common feature of the intron in prokaryotic species but has also been observed in the organelle genomes, deriving from endosymbiotic events [90]. The reverse transcriptase domain is usually observed in group II introns involved in intron mobility. Further dedicated work must verify the capacity of these putative group II introns in *H. lacustris* to induce self-splicing and possibly move to other genes. Finally, the *psaA* gene is present in four fragments scattered in the plastidial genome and on different strands, perhaps requiring a trans-splicing mechanism as described in other species, such as *C. reinhardtii* [91].

*H. lacustris* mitochondrial genome was entirely reconstructed as having 145 kb size (Supplementary dataset 5, Supplementary Table 8), the largest known among Chlorophyta, and even larger compared to the previous report, which suggested a 124.65 kb size for *H. lacustris* mitochondrial genome [34]. Like the chloroplast case, most of the mitochondrial genome is composed of repetitive DNA. Among the genes in the mitochondrial genome, genes encoding for 23 fragments of rRNAs and three tRNAs were identified. It is important to note that in the closely related species *C. reinhardtii*, rRNA genes were found broken into several pieces that interspersed with one another and with protein- and tRNA-coding regions [92]: it cannot be excluded that a similar situation is also happening in the case of 12S and 16S rRNA of *H. lacustris* mitochondrial genome. Other genes encoded by the *H. lacustris* mitochondrial genome are subunits of the electron transport chain, particularly 6 subunits of Complex I and 2 genes for Complex III and IV subunits (cob and cox1, respectively).

### 3.7 Differential gene expression in stressing conditions

**Fig. 5:**
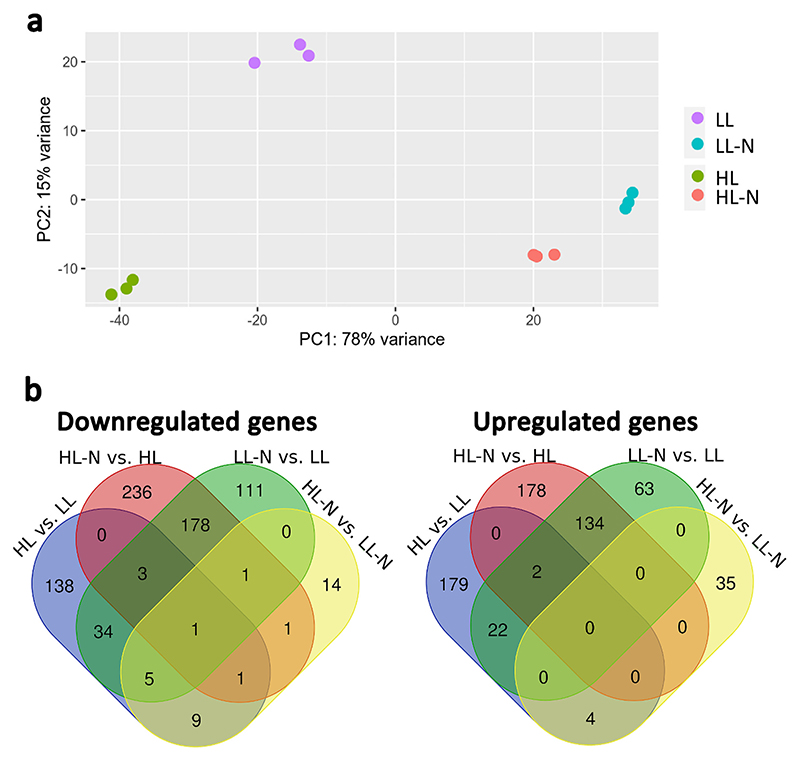
RNA-seq analysis of *Haematococcus lacustris* cells grown in low light or high light in the presence or absence of nitrogen starvation. a) Principal component analysis (PCA) of the different samples analyzed (*n* = 3): RNA extracted from *H. lacustris* cells grown in low light without nitrogen starvation (LL), high light without nitrogen starvation (HL), low light in nitrogen starvation (LL-N), high light in nitrogen starvation (HL-N). b) Venn diagrams of differentially expressed genes in the growth conditions herein analyzed.

RNA-seq results were analyzed to identify the genes in *H. lacustris* that were differently expressed in the growth conditions herein applied (HL, LL, HL-N, and LL-N, Fig. 5a) to evaluate the effects of stresses, such as high irradiance and nitrogen starvation on gene expression. Nevertheless, it is important to point out that changes in the mRNA levels in some cases do not imply changes in the final gene product. In addition, changes in the mRNA levels could also be related to other cell processes not directly correlated to the stressing conditions herein applied. The genes differentially expressed in the comparisons HL vs. LL, LL-N vs. LL, HL-N vs. HL, and HL-N vs. LL-N were respectively 398, 554, 735, and 71. The relatively low number of differentially expressed genes in HL-N vs. LL-N suggests that nitrogen starvation is a predominant stressing condition compared to high irradiance in the conditions herein tested, inducing already a strong regulation of transcription (Supplementary Fig. 5). Accordingly, a significant fraction of genes differentially expressed in LL-N vs. LL was also differentially expressed in HL-N vs. HL (Fig. 5b), suggesting a potential role for these genes in nitrogen starvation. Only a few genes were differentially expressed based on the light intensity at which the cells were exposed during growth: 4 and 9 genes were respectively upregulated or downregulated in both HL vs. LL and HL-N vs. LL-N conditions but not in response to nitrogen starvation. These genes encode mainly for chlorophyll-binding proteins and chitinase II enzyme (downregulated) or a fasciclin-like (FAS1) protein and a G-protein coupled receptor-related (upregulated). Fasciclin-like proteins are usually found in cell walls [93]. Other FAS1 proteins could be found specifically upregulated in HL vs. LL or HL-N vs. LL-N, while these genes were downregulated due to nitrogen starvation (HL-N vs. HL and LL-N vs. LL). Only in one case (g12213) was a predicted FAS1 protein upregulated in cells under nitrogen starvation (HL-N vs. HL).

**Fig. 6:**
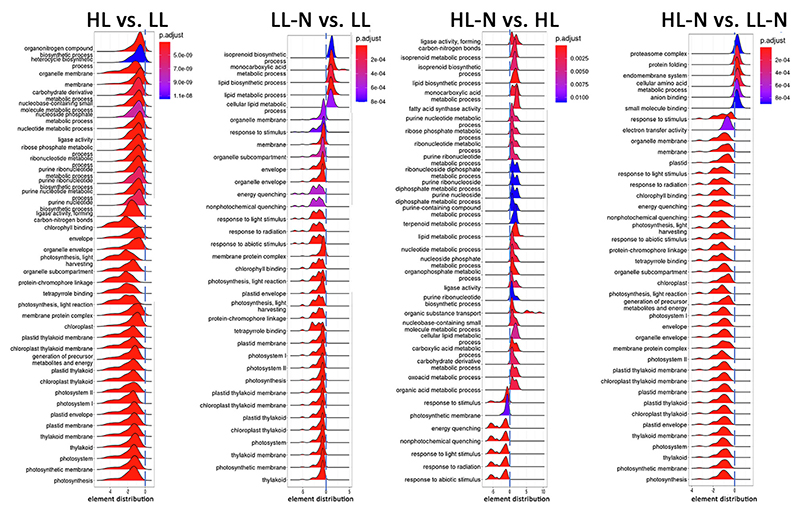
Gene Ontology enrichment of differentially expressed genes in nitrogen starvation and/or high irradiance. Gene Ontology enrichment of differentially expressed genes in cells grown under the conditions herein tested, being high light (HL), low light (LL), high light in nitrogen starvation (HL-N) or low light in nitrogen starvation (LL-N), is reported with negative distribution for downregulated genes and positive distribution for upregulated genes. Each plot features the log2 fold change (log2FC) along the x-axis, and the y-axis depicts the frequency of log2FC values for each gene within the gene sets of each GO term. P-adjusted values for the different groups is reported with blue to red gradient color according to the scale reported for each condition. (For interpretation of the references to color in this figure legend, the reader is referred to the web version of this article.)

GO classification of differentially expressed genes was used to generate functional enrichment of the expression dataset (Fig. 6). In the case of genes differentially expressed as a response to the irradiance applied during growth (HL vs. LL and HL-N vs. LL-N), the most significant regulation was observed in the downregulation of genes involved in the light phase of photosynthesis, thylakoid membrane formation, and chloroplast biogenesis: this is in line with the reduced chlorophyll to the carotenoid ratio observed in HL grown cells compared to LL, which was further decreased under nitrogen limitation (HL-N vs. LL-N) (Supplementary Table 5). Similar downregulation of genes involved in the photosynthetic activity and chloroplast biogenesis was observed due to nitrogen starvation (LL-N vs. LL and HL-N vs. HL), where indeed chlorophyll content and photosynthetic activity of the cells were strongly reduced [9,11]. In the case of cells exposed to nitrogen starvation (LL-N vs. LL and HL-N vs. HL), upregulation of genes involved in lipid biosynthesis and terpene metabolism was observed. Lipids are the main macromolecule class accumulated by several microalgae species under nitrogen starvation, with their nitrogen composition highly reduced compared to other biomass constituents 49,50. At the same time, terpenes are secondary metabolites, which include carotenoids and astaxanthin, with different possible roles in the cells, such as ROS scavenging and protection from photooxidative stress. In HL-grown cells, nitrogen starvation led to an upregulation of genes involved in nucleotide metabolism (HL-N vs. HL, Fig. 6), being essentially related to ATP and purine metabolism: purine has been previously reported as a possible source of nitrogen for cells in nitrogen starvation [94]. Genes encoding for xanthin/uracil permease are among the genes with the highest upregulation in both HL-N vs. HL and LL-N vs. LL cells (Supplementary Table 9). Similarly, two SLC5sbd_DUR3 encoding genes (g7818 and g2542) were strongly upregulated in cells under nitrogen starvation in both HL and LL conditions (HL-N vs. HL and LL-N vs. LL): DUR3 belongs to the solute carrier 5 (SLC5) transporter family, and it is involved in urea transport [95], where urea is one of the products being released upon purine degradation. Downregulation of genes involved in ATP biosynthesis in HL-grown cells compared to LL is consistent with the reduced chlorophyll content and, thus, reduced photosynthetic activity in HL cells compared to LL. In the case of HL-N vs. LL-N comparison, upregulation of genes involved in proteasome activity, amino acid metabolism, and protein folding suggested that the strong stress induced by exposure to high light and nitrogen starvation led to the rearrangement of protein content in the cells with degradation of specific protein targets.

### 3.8 Identification of genes involved in key metabolic pathways and their differential expression

The functional annotation of the *H. lacustris* genome allowed for the identification of genes coding for the key enzymes involved in the different metabolic pathways of the cell and their changes in the other growth conditions, which are briefly presented below.

#### 3.8.1 Photosynthesis

All the Photosystem II core subunits usually found in green algae and land plants [96] were identified even in *H. lacustris* encoded in the nuclear or plastid genomes.

In the case of Photosystem I complex, all core subunits were identified except for PsaM, PsaN, and PsaX: while PsaM and PsaX were also not identified in the case of other green algae such as *C. vulgaris* or *C. reinhardtii* [97], PsaN is a PSI subunit usually found in green algae and land plants, but not in cyanobacteria. PsaN was identified in the lumenal side of PSI being involved in the interaction with plastocyanin [98]: Interestingly, no PsaN could be found in the moss *Physcomitrium patens*, revealing a complex evolutionary profile of this subunit [99].

Different chlorophyll-binding Light Harvesting Complexes (LHC) were identified in *H. lacustris*, being predicted as Lhca or Lhcb subunits, antenna complexes, respectively, for PSI or PSII. Consistent with the case of *C. reinhardtii*, no gene coding for LHCB6 (CP24) or LHCB3 proteins was found, supporting the hypothesis that these PSII antenna proteins occur only in land plants [100]. Both the LHC-like subunits PSBS and LHCSR subunits, involved in the photoprotective mechanism known as non-photochemical quenching (NPQ), can be found in *H. lacustris*: when NPQ is induced, a significant portion of the light energy absorbed is dissipated as heat to prevent saturation of the light phase of photosynthesis. PSBS is the thylakoid membrane protein responsible for NPQ induction in vascular plants. At the same time, LHCSR subunits have been reported as the critical subunits for NPQ in the model organism for green algae, *C. reinhardtii* [101,102]. In the case of *H. lacustris*, PSBS and LHCSR proteins are encoded by two (g1838 and g8651) and three genes (g11342, g2272, g288). Differently from *C. reinhardtii*, where LHCSR subunits are strongly upregulated in cells exposed to HL [101], g11342 and g288 are not differentially expressed in tested growth conditions, while the g2272 gene is instead down-regulated upon exposure to HL (Supplementary Table 10). In the case of *psbs*, the gene g8651 is strongly upregulated in cells under nitrogen starvation and low light (LL-N vs. LL) and slightly upregulated as a response to high irradiance in HL vs. LL and HL-N vs. LL-N cells (Supplementary Fig. 6). PSBS expression profile is different compared to the case of *C. reinhardtii*, where *psbs* is only transiently expressed in UV or HL conditions [103–105]: in the case of *H. lacustris*, the *psbs* gene is always expressed but upregulated by nitrogen starvation and HL (Supplementary Table 10). These results suggest a different regulation of NPQ in *H. lacustris* compared to *C. reinhardtii*, even if additional confirmatory experiments should verify the role of LHCSR and PSBS.

Genes encoding protein subunits reported in *C. reinhardtii* to be involved in alternative chloroplast electron transport pathways are present in the *H. lacustris* genome (Supplementary Table 11). PGR5 (Proton Gradient Regulator)-like proteins [106] [107] are involved in cyclic electron flow: PGR5-like proteins in *H. lacustris* are encoded by g449 and g454 genes, which are not differentially expressed in cells under different growth conditions. PTOX (Plastid Terminal Oxidase) is an oxidase involved in chlororespiration [108,109] but this enzyme is also linked to carotenogenesis and astaxanthin production, being involved in the redox reaction of phytoene desaturase and/or ζ-carotene desaturase [110,111]. In the case of PTOX, two genes, g11573 and g5100, were identified with a complex expression profile. The gene g11573 is upregulated due to cells exposure to HL only under nitrogen-replete conditions (HL vs. LL) but not under nitrogen starvation (HL-N vs. LL-N), while downregulation of this gene could be observed as a response to nitrogen starvation, especially in cells exposed to HL (HL-N vs HL). In the case of g5100, a slight upregulation could be observed under nitrogen starvation (LL-N vs. LL) and (HL-N vs. HL). The presence of two genes coding for a plastid terminal oxidase is consistent with previous reports [15,110,112].

In the case of the dark phase of photosynthesis and carbon fixation, all subunits previously reported to be involved in this pathway have been identified (Supplementary Table 12). Most genes involved in carbon fixation were downregulated in HL vs. LL cells but were instead up-regulated by nitrogen starvation in HL-grown cells (HL-N vs. HL). The upregulation of genes involved in carbon fixation in HL-N cells is consistent with the increased photosynthetic activity on a chlorophyll basis observed in these conditions compared to HL-grown cells [113]. It is possible to speculate that the increased availability of enzymes involved in carbon fixation in the extremely stressing condition of high irradiance and nitrogen starvation (HL-N) might be a strategy to regenerate the ADP and NADP^+^ cofactors required to desaturate the photosynthetic apparatus to mitigate photooxidative stress. However, it is essential to note that the enzymes downregulated in HL vs. LL and upregulated in HL-N vs. HL grown cells as transketolase (g11095) and ribulose-phosphate 3-epimerase (g8002) and fructose-1,6-bisphosphatase (FBP, g3673) are also involved in pentose phosphate pathway to produce NADPH. Their downregulation in HL vs. LL cells could be related to the saturation of NADPH, making the light phase of photosynthesis in cells grown at high irradiance. In contrast, their upregulation in HL-N vs. HL cells could be associated with the requirement of reducing power for lipid biosynthesis (see below).

#### 3.8.2 Terpenes and carotenoid biosynthesis

**Fig. 7:**
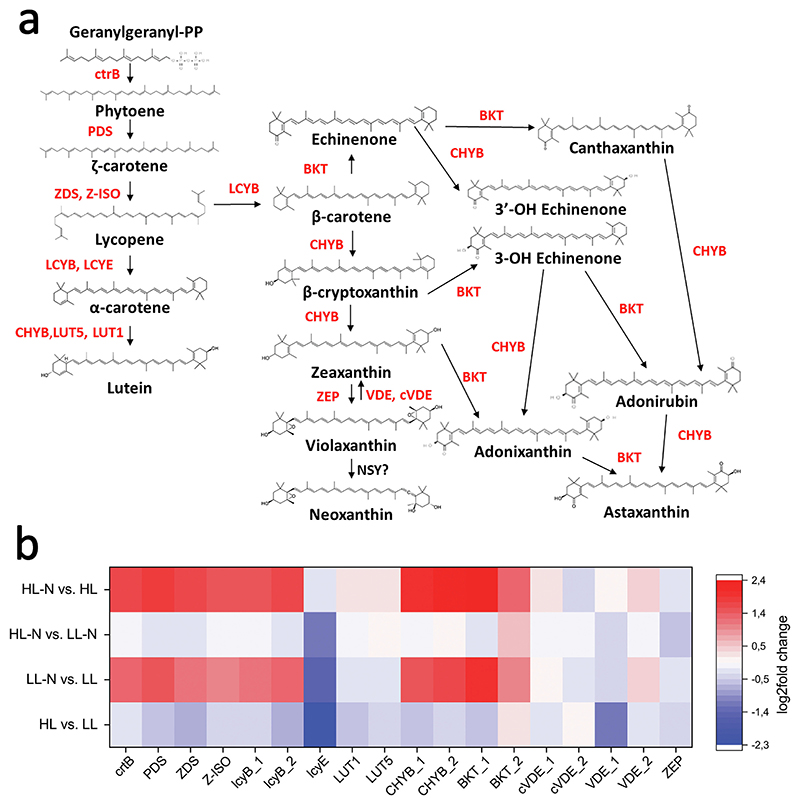
Carotenoid biosynthetic pathway genes in *Haematococcus lacustris* and their expression in different growth conditions. a) Schematic carotenoid biosynthetic pathway. b) log2fold change of gene expression for the different genes reported in (a) in cells grown in high light (HL), low light (LL), high light in nitrogen starvation (HL-N), or low light in nitrogen starvation (LL-N). Only major carotenoids are indicated. Names of enzymes are reported. CtrB: phytoene synthase, PDS: phytoene desaturase, ZDS: ζ-carotene desaturase, CRTISO: carotenoid isomerase, LCYB: lycopene β-cyclase, LCYE: lycopene ε-cyclase, CHYB: Carotene β-hydoxylase, CHYE: carotene ε-hydoxylase, BKT: carotene β-ketolase, ZEP: zeaxanthin epoxidase, VDE: violaxanthin de-epoxidase, cVDE: Chlamydomonas-like violaxanthin deepoxidase, NSY: neoxanthin synthase.

Terpenes and carotenoids are synthesized using isopentyl-diphosphate (IPP) as a precursor. Biosynthesis of IPP and its isomer dimethylallyl-diphosphate (DMAPP) occurs in algal cells by two independent pathways: the mevalonate pathway in the cytosol and the Methylerythritol 4-phosphate (MEP) pathway in chloroplasts. Consistent with previous reports, only enzymes involved in the MEP pathway were found in the *H. lacustris* genome (Supplementary Fig. 7). The loss of the MVA pathway is a standard feature for green algae, while this pathway can be found in land plants, eukaryotic heterotrophs, or other algae genera [114,115]. As reported in Supplementary Fig. 7, upregulation of essential genes involved in IPP biosynthesis as *dxs, ispG, ispH*, and *ggps* could be observed due to nitrogen starvation, especially in HL-grown cells (HL-N vs. HL), also the condition inducing the strongest accumulation of the carotenoid astaxanthin. Carotenoid biosynthetic genes were identified in the *H. lacustris* genome (Fig. 7). Each of the genes involved in carotene and xanthophyll biosynthesis was found in a single copy except for the enzymes LCYB (lycopene-β-cyclase), BKT (β-carotene ketolase), CHYB (β-carotene 3-hydroxylase) and VDE (violaxanthin-depoxidase) since all those were encoded by two gene copies (Supplementary Table 13). BKT and CHYB (called respectively also crtW and crtZ) are the key enzymes for astaxanthin biosynthesis, being involved respectively in the catalysis of the ketolating and hydroxylating reactions that allow the conversion of β-carotene into astaxanthin [116]. The presence of multiple genes coding for the BKT enzyme is consistent with previous reports: three BKT proteins encoded by six genes were suggested in the case of the *H. lacustris* genome published by Luo and coworkers [29], while in the newly assembled genome herein reported only two genes (g1780 and g8702) were identified as *bkt* genes (Supplementary Fig. 8). Most of the genes encoding for enzymes involved in carotenoid biosynthesis were upregulated in cells under nitrogen starvation (LL-N vs. LL and HL-N vs. HL), except for the enzymes involved in lutein biosynthesis (LCYE, LUT1, LUT5) and the xanthophyll cycle (VDE, cVDE, and ZEP), which were not differently expressed. Exposure to high light (HL vs. LL and HL-N vs. LL-N) caused a minimal effect on the expression of carotenoid biosynthetic enzymes: only a significant downregulation of LCYE in HL vs. LL cells was observed, even if it is worth noting that one of the gene encodings for BKT enzyme, g8702, showed an increased expression when cells were exposed to high light (Fig. 7). These data support the finding of the highest accumulation of astaxanthin in HL-N cells compared to other growth conditions. In the presence of both nitrogen starvation and high irradiance stress, the cell metabolism is redirected toward strong astaxanthin accumulation by a strong upregulation of terpenes and carotenoids biosynthetic genes.

#### 3.8.3 Glycolysis, gluconeogenesis, and TCA cycle pathways

Complete sets of genes encoding for glycolysis and gluconeogenesis were retrieved in the *H. lacustris* genome. Most of these genes were not differentially expressed in the conditions herein tested except for genes coding for a fructose-1,6-bisphosphatase I and a pyruvate phosphate dikinase, both specifically involved in gluconeogenesis and upregulated in HL vs. LL [117], a phosphoglycerate kinase, involved in both glycolysis and gluconeogenesis upregulated in HL vs. LL cells and in HL-N vs. HL cells, and a pyruvate kinase, specific for glycolysis, upregulated in HL-N vs. HL cells (Supplementary Fig. 9). It is thus possible to speculate that the upregulation of fructose-1,6-bisphosphatase I and pyruvate phosphate dikinase in HL vs. LL cells redirect the carbon flow toward carbohydrates production, while in nitrogen starvation and high irradiance (HL-N vs. HL) the upregulation of pyruvate kinase boost glycolysis with the production of reducing power, ATP and precursors for lipid biosynthesis as pyruvate. Accordingly, strong upregulation of E1 (g10979 and g6781) and E2 (g4387) subunits of pyruvate dehydrogenase was observed in HL-N vs. HL cells, being this enzyme involved in pyruvate oxidation with the formation of acetyl-Coa for lipid biosynthesis. Further experimental evidence is required to support this hypothesis.

Genes encoding for enzymes involved in the TCA cycle were identified in *H. lacustris* (Supplementary Fig. 9). Among these genes, differential expression was observed only in the case of a gene encoding for an isocitrate dehydrogenase (g4258), which is strongly upregulated in HL-N vs. HL cells and downregulated in HL vs. LL cells. Slightly increased expression of isocitrate dehydrogenase enzyme was also observed in cells under LL-N vs. LL conditions, although with only a log2 fold change of 1.16 (Supplementary dataset 1). Interestingly, isocitrate dehydrogenase was reported as being upregulated in cells under nitrogen starvation in different species of cyanobacteria and microalgae [118,119]. This enzyme catalyzes the decarboxylation of isocitrate to produce 2-oxoglutarate, which is then involved in glutamate biosynthesis.

Critical enzymes for the glyoxylate cycle were also identified as isocitrate lyase (g8616), malate synthase (g7123), and malate dehydrogenase (g5782 and g5817). The isocitrate lyase-encoding gene (g8616) was upregulated in HL vs. LL cells but not differentially expressed in other conditions. At the same time, malate synthase (g7123) was downregulated in cells under nitrogen starvation (LL-N vs. LL and HL-N vs. HL). Upregulation of the glyoxylate cycle in HL is consistent with the proposed increase of gluconeogenesis in cells grown at high irradiance; oxalacetate is produced by the glyoxylate cycle and used as a precursor for carbohydrate biosynthesis. Similar upregulation of glyoxylate in cells exposed to HL conditions was also observed in the case of *C. vulgaris* [87]. The glyoxylate cycle has been reported to be in ancestral peroxisomes of *C. reinhardtii* [120], but its localization should be further investigated in *H. lacustris*.

#### 3.8.4 Nitrogen assimilation

Eleven genes encoding for nitrate transporters were identified. Most of these genes were downregulated in response to nitrogen starvation (LL-N vs. LL) or high irradiance (HL vs. LL), while the combined effect of both stresses did not further affect their expression. An exception is posed by g2496 and g2457 genes, which are downregulated in HL-N compared to HL cells (Supplementary Table 14). Two genes encoding for putative nitrate reductase (g2446 and g2501) were identified, and both were downregulated in cells under nitrogen starvation (HL-N vs. HL and LL-N vs. LL). In the case of nitrite reductase, the five putative identified genes (g125, g2525, g49, g6067, g8151) were not differentially expressed under the different growth conditions except for g125 and g49 genes being downregulated by nitrogen starvation (LL-N vs. LL). Being nitrate the only external nitrogen source in the herein tested conditions, the low availability of nitrate in nitrogen starvation conditions leads to the downregulation of the enzymes involved in its assimilation. One of the critical enzymes involved in ammonium assimilation, glutamine synthase, is putatively encoded by two genes in *H. lacustris* (g10340, g4053), which were upregulated under nitrogen starvation (LL-N vs. LL and HL-N vs. HL) but downregulated upon exposure to HL (HL vs. LL). In the case of glutamate synthase, two encoding genes were identified, g3249 and g5943, with the former being upregulated in HL-N vs.-HL cells. A gene for glutamate dehydrogenase (g2149), catalyzing the direct formation of glutamate from ammonium and oxoglutarate, was also identified but not differentially expressed in the growth conditions herein tested.

#### 3.8.5 Lipid biosynthesis

Genes encoding critical lipid metabolism proteins were identified in the *H. lacustris* genome; most were upregulated in HL-N vs. HL cells (Supplementary Table 15). Several enzymes involved in fatty acid biosynthesis, Acetyl-CoA carboxylase components, Malonyl-CoA: ACP transacylase, and the different subunits of Fatty acid synthase Type II were upregulated in HL-N vs. HL cells. In contrast, genes involved in glycerol-3-phosphate (G3P) and TAG packaging were not differentially expressed. Intriguingly, among the highly upregulated genes in LL-N vs. LL cells, it was possible to identify a gene putatively encoding for acetyl-CoA synthetase (ACS, g11655). This enzyme is involved in the pyruvate dehydrogenase bypass pathway by which acetyl-CoA, a substrate for fatty acid biosynthesis, is produced by glycolytic pyruvate through the intermediates acetaldehyde and acetate [121]. In *A. thaliana*, mutations in the *acs* genes caused a substantial reduction in plant fitness [121].

In the case of enzymes involved in fatty acid degradation, only genes encoding for Acyl-CoA dehydrogenase were identified as differentially expressed, with g1764, g1716, and g4556 being downregulated in HL vs. LL or LL-N vs. LL cells. According to the results obtained, it is possible to suggest that nitrogen starvation boosts fatty acid biosynthesis by upregulating anabolic enzymes when cells are exposed to both nitrogen starvation and high irradiance. In contrast, downregulation of catabolic enzymes occurs when only one of the two stresses is present.

In *H. lacustris*, astaxanthin accumulation in stressing conditions is notoriously linked to lipid biosynthesis and the formation of lipid droplets in the cytosol [14,116,122]. In the case of *H. lacustris*, several genes were annotated as Plastid-lipid Associated proteins (PAP/fibrillin), MLDP (major lipid droplet protein), or caleosin (Supplementary Table 16). Differently, no putative oleosin could be found in the *H. lacustris* genome, oleosin being the main lipid-droplet binding protein in higher plants [123]. PAP/fibrillin proteins have been reported to be involved in forming lipid droplets in microalgae [124]: surprisingly, none of these genes were differentially expressed under the condition herein tested. Differently, among the two genes (g2545 and g8) annotated as MLDP (major lipid droplet protein), the primary lipid droplets-associated proteins reported in green algae [125], g2545 was upregulated under nitrogen starvation (LL-N vs. LL and HL-N vs. HL) and downregulated upon exposure to HL (HL vs. LL), suggesting its specific role in lipid droplets formation is triggered by nitrogen starvation. It is important to note that both MLDP putative genes identified in the *H. lacustris* genome share a significant identity with the HOGP (Haematococcus Oil Globule Protein) protein previously isolated from astaxanthin-rich lipid droplets in *H. lacustris* [122] (Supplementary Fig. 10). In the case of genes annotated as caleosin-like proteins in the *H. lacustris* genome (g13433 and g7787), one gene was strongly upregulated upon exposure to HL (HL vs. LL) but downregulated by nitrogen starvation (HL-N vs. HL). In contrast, the other gene (g13433) was upregulated in cells under nitrogen starvation in both low light or high light conditions (LL-N vs. LL and HL-N vs. HL). Caleosin are calcium-binding proteins that can be found in multicellular plants and green algae, frequently described as lipid droplets-associated proteins [126,127].

## 4 Discussion

The development of advanced tools for genetic engineering, such as genome editing or common syntax for synthetic biology applications, requires the availability of high-quality genome assembly and functional annotation. Here, we provide a novel genome of *H. lacustris* assembled in 32 scaffolds (Fig. 1) containing 91 % of the 151 Mb genome of *H. lacustris* with a scaffold N50 of 4 Mb (Table 1). The generated assembly represents a >4-fold improvement in contiguity compared with the previously published assembly of *H. lacustris*, and its scaffold N50 is in line with the case of *Chlamydomonas reinhardtii*, the model organism for green algae (Table 1). In addition, putative telomeric repetitive motifs were found at the ends of all the scaffolds herein assembled (Supplementary Table 4). A key feature observed in the *H. lacustris* genome is the average larger size of Simple Sequence Repeats (SSRs) in coding sequences (Table 2), despite the reduced number of genes with SSRs (Table 2). The function of SSRs in coding sequences has been extensively investigated in the human genome, mainly associated with human diseases. At the same time, little information is available in the case of plants or algae genomes. Previous reports speculated that eukaryotes incorporating more DNA repeats might provide a molecular device for faster adaptation to environmental stresses [128,129]. *H. lacustris* cells are among the microalgal cells with the most evident physiologic responses upon exposure to stresses, such as cell expansion, transition to a non-motile cell state, and substantial accumulation of carotenoids. It is thus possible to speculate that the evolutionary origin of *H. lacustris* caused the development of multiple mechanisms in this species to mitigate the consequences of exposure to stress conditions, including the accumulation of highly repetitive DNA sequences.

Another key feature of the *H. lacustris* genome is its diploid genome configuration. The quality of the previous genome assembly reported [29–31] did not allow us to decipher the ploidy of this species. It is also interesting to note that the *H. lacustris* genome size identified in this work (151 Mb) is essentially half of the genome size estimated in the most recent *H. lacustris* genome assembly [31] but in line with the genome size proposed by Morimoto and coworkers (171 Mb) [30]. The two-fold genome size of *H. lacustris* proposed by Bian and coworkers may be related to the presence of two copies of the *H. lacustris* genome. This hypothesis is consistent with the collinearity analysis between the genome scaffolds reported in this work and the scaffolds reported by Bian and coworkers. As reported in Fig. 2, for each scaffold assembled it is possible to notice a double alignment with the sequences reported by Bian and coworkers, as expected because the diploidy of *H. lacustris* genome was not considered in the previous genome draft [31]. Here, the diploid configuration of the *H. lacustris* genome was validated by two independent approaches based respectively on in silico evaluation of SNPs distribution and analysis of SNPs propagation in cell progeny, demonstrating the presence of a second copy of the *H. lacustris* genome in the cells. It is important to note that the three *Haematococcus* strains herein mentioned were isolated from different sites, being K-0084 was isolated in Sweden, while NIES-144 and SAG192.80 were isolated respectively in Japan and in Germany according to the culture collections where these strains are available: even if these strains are phylogenetically related (Fig. 4), it cannot be excluded that the different genetic features herein retrieved, including the diploid genome configuration could be related to strain specific features. The presence of a diploid genome could be associated with a possible capacity to better tolerate the onset of mutations, even in essential genes, and the feasibility of having a higher genetic variability. In general, a positive correlation between the increased ploidy level and resistance to abiotic stress was reported in the case of plant genomes, even if this view needs further evidence to be supported [130]. The presence of a diploid genome should be carefully considered in developing biotechnological tools for *H. lacustris* genetic engineering: the randomness of site-specific mutation approaches may result in the modification of only one allele, with different possible outcomes for the resulting phenotypes. Moreover, the possible induction of sexual replication in *H. lacustris* cells, even if not fully understood and reproducible in laboratory conditions, may occur in some specific conditions with a potential loss of heterologous DNA sequences introduced in only one genome copy. In the case of several diatom species, such as *Phaeodactylum tricornutum*, the occurrence of microalgae with diploid genes is common, thus requiring dedicated molecular biology tools to introduce mutations or novel genes [131,132].

The chloroplast and mitochondrial genomes of *H. lacustris* are extraordinarily large, up to 1.42 Mb and 145 kb, respectively. The large size of the plastid and mitochondrial genomes is mainly related to non-coding and repetitive sequences, as previously reported [32–34]. A peculiar feature of the plastid genome is the presence of type-2 introns encoding for intron maturase, reverse transcriptase, or deoxyuridine 5′-triphosphate nucleotide hydrolase enzymes. These findings suggest the possible self-splicing activity of these introns, but their activity, functions, and evolutionary origin need to be further verified with dedicated work.

The functional annotation of the newly assembled genome and the differential expression analysis of the annotated genes in the growth conditions herein tested allowed us to highlight critical genes involved in *H. lacustris* cell functions and adaptations to stress conditions. The key enzymes involved in carotenogenesis and ketocarotenoid biosynthesis were identified, with BKT and CHB enzymes being encoded by two gene copies each. The expression profile of genes involved in astaxanthin biosynthesis revealed an upregulation induced by nitrogen starvation, which, in our experimental set-up, was the condition that caused the strongest astaxanthin accumulation. Similarly, the genes involved in the biosynthesis of terpenes precursors as *dxs, ispG, ispH*, and *ggps* were upregulated by nitrogen starvation. Another enzyme previously reported as being involved in carotenoid biosynthesis is the plastid terminal oxidase PTOX, which uses the electrons released by the desaturating reactions occurring in this pathway. Previous work suggested the presence of multiple genes (*PTOX1* and *PTOX2*) encoding for this enzyme [15,110,112]: here, we confirm the presence of two PTOX genes (g11573 and g5100), which are upregulated by high irradiance or nitrogen starvation, respectively. The high light-induced upregulation of only one of the two *PTOX* genes is consistent with a previous report [15], where, however, the effect of nitrogen starvation was not studied. Our findings suggest a differential contribution for the two *PTOX* genes of *H. lacustris* when exposed to high light or nitrogen starvation. In contrast, the potential differential expression of these genes induced by other stresses should be further investigated.

The HL treatment herein applied only partially triggered astaxanthin biosynthesis (Supplementary Table 5): previous work demonstrated that HL exposure triggers photoprotective mechanisms such as NPQ and lipid biosynthesis [14,133]. Here, it was possible to observe in HL vs. LL cells an upregulation of one of the critical proteins involved in NPQ in land plants, PSBS (g8651), whose role in the case of *H. lacustris* needs to be further investigated. The combined exposure to high light and nitrogen starvation (HL-N), in the conditions herein applied, was the most efficient condition to induce astaxanthin biosynthesis (Supplementary Table 5), with low nitrogen availability generating the strongest effect in terms of differential gene expression (Fig. 6). Nitrogen starvation caused the downregulation of nitrate transporters and genes encoding for nitrate and nitrite reductase, while glutamate synthase and glutamine synthase were upregulated. It is important to note that a gene encoding for an isocitrate dehydrogenase was strongly upregulated in cells under nitrogen starvation (HL-N vs. HL): one of the products of the reaction catalyzed by this enzyme is 2-oxoglutarate whose accumulation was previously correlated with astaxanthin production in *H. lacustris* [13]. This observation is consistent with HL-N as the condition inducing the strongest astaxanthin accumulation. Nitrogen starvation in both HL and LL conditions caused a decrease in chlorophyll, consistent with previous observations [11] and with the downregulation of genes involved in tetrapyrrole biosynthesis. Interestingly, upregulation of several enzymes involved in carbon fixation was observed in HL-N vs HL cells: this finding is consistent with previous observations [134,135] about an increase in Rubisco activity when *H. lacustris* cells are exposed to oxidative stress. It was hypothesized that an increased activity of carbon fixation enzymes when chlorophyll content is strongly decreased, consisting in the redirection of carbon flow toward glycolysis, TCA, and pentose phosphate pathway to produce the ATP and reducing the power required for carbon fixation resulting in astaxanthin and lipid biosynthesis as a carbon sink [135]. The differential gene expression herein investigated supports this finding, with upregulation in HL-N vs. HL cells of genes involved in glycolysis, such as pyruvate kinase and phosphoglycerate kinase, and in the conversion of pyruvate to acetyl-CoA (pyruvate dehydrogenase), which is the precursor for both TCA cycle and lipid biosynthesis. Moreover, enzymes potentially involved in both the pentose phosphate pathway and carbon fixation, including transketolase (g11095), ribulose-phosphate 3-epimerase (g8002), and fructose-1,6-bisphosphatase (FBP, g3673) were upregulated in HL-N vs. HL cells.

Astaxanthin biosynthesis is strictly related to lipid biosynthesis and lipid droplet formation [14,136,137]. Several enzymes involved in fatty acid biosynthesis were upregulated in HL-N vs. HL cells. In contrast, downregulation of enzymes involved in fatty acid degradation was observed in HL vs. LL cells: these differential expression patterns suggest de novo fatty acid biosynthesis and reduced fatty acid degradation as the two possible mechanisms for increased lipid accumulation in cells exposed respectively to nitrogen starvation and high light. Formation of lipid droplets requires the interaction between lipids and lipid-binding proteins: PAP-fibrillin, MLDP, and caleosins encoding genes could be identified in the newly assembled *H. lacustris* genome, while no oleosin protein could be observed. Oleosins are the major lipid droplet-associated proteins found in higher plants, but their conservation was also reported in the case of some green algae such as *C. reinhardtii* and *Volvox carterii* [138]. The absence of oleosin in *H. lacustris* or other green algae such as *C. vulgaris* suggests an evolutionary pattern for these proteins, which only in land plants became the main proteins involved in lipid droplet formation. It is important to note that a strong upregulation of genes encoding respectively for an MLDP (g2545) previously isolated in astaxanthin-rich lipid droplets [122] (Supplementary Fig. 10) or for a caleosin-like protein was observed respectively due to nitrogen starvation or high irradiance, suggesting that lipid droplets formation under high irradiance and/or nitrogen starvation involve a proper tuning of lipid droplets packaging proteins.

In conclusion, the assembly and functional annotation of the *H. lacustris* genome allowed the identification of potential targets for biotechnological manipulation of this organism to improve astaxanthin biosynthesis. The genomic and transcriptomic data herein described will enable us to draw a model for *H. lacustris* responses to different stressing conditions, leading to astaxanthin biosynthesis. However, it should be considered that further events at translational or post-translational levels could also take place, affecting the highlighted metabolic pathways. Overexpression of genes involved in photoprotection and ROS scavenging, using inducible promoters, could be a strategy to mitigate the photooxidative stress, allowing higher biomass production before inducing astaxanthin biosynthesis. Alternatively, the key enzymes involved in terpenes and lipid biosynthesis and packaging upregulated in stress conditions could be triggered in optimal growth conditions to boost astaxanthin production even in the absence of stresses.

## Supplementary Material

Supplemental Tables 1-8 and Figures 1-10

Supplemental Tables 9-16

## Figures and Tables

**Table 1:** Statistics of *Haematococcus lacustris* genome compared to previous assemblies and the *Chlamydomonas reinhardtii* case.

	*Haematococcus lacustris* (this work)	*Haematococcus lacustris* [30]	*Haematococcus lacustris* [31]	*Chlamydomonas reinhardtii* CC503 [85]	*Chlamydomonas reinhardtii* CC- 4532 [86]
Total assembly length (bp)	150,042,165	171,794,631	309,350,987	111,098,438	114,631,715
Total scaffolds length (bp)	137,192,742	163,427,503	291,198,747	108,896,203	112,319,453
Number of scaffolds/chromosomes	32	6224	67^a^	17^b^	17^b^
Scaffolds N50 (bp)	4,010,071	38,941	942,600	7,783,580	6,954,842
Scaffold average length (bp)	4,287,273.19	26,257.63	4,346,249	6,405,659	6,607,026.6
Longest scaffold (bp)	9,907,970	215,986	21,607,000	9,730,733	9,952,739
Shortest scaffold (bp)	1,735,284	1671	5000	1922,86	3,682,160
Number of gaps	930	31,341	2830	1441	63
Gaps size (bp)	93,000	44,043,566	1,399,000	4,055,092	928,517
Contigs in scaffolds	962	37,565	2897	1476	80
Remaining contigs	839	3469	753	36	40
Remaining contig total length (bp)	12,849,423	8,367,128	18,152,240	2,202,235	1,716,047

a Among the 67 scaffolds assembled in Bian et al. 2023 the 32 scaffolds longer than 1 Mb were associated with 32 putative chromosomes.

b In the case of *Chlamydomonas reinhardtii* 17 chromosomes were identified with a corresponding number of scaffolds being anchored by genetic mapping.

**Table 2:** Analysis of simple sequence repeat in CDS in *Haematococcus lacustris, Chlamydomonas reinhardtii* and *Volvox carteri*. The number of genes with Simple Sequence Repeat (SSRs) in the coding sequence (CDS) is reported for the three algal species herein investigated. The length of these repeating motifs, which can range from 1 to 5 bases, is reported as “block size”. The number of genes of different block sizes and the average length in bp of repeated sequences for the different block sizes are also reported.

		*Haematococcus* *lacustris*	*Chlamydomonas* *reihnarditii*	*Volvox* *carteri*
Number of genes	Number of genes with SSR CDS	727 (5.2 %)	3272 (18.4 %)	2090 (14.7 %)
	Total number of genes	13,946	17,741	14,247
	Block size	*Haematococcus* *lacustris*	*Chlamydomonas* *reinhardtii*	*Volvox* *carteri*
Number of predicted genes with SSR	1	2	0	0
	2	0	2	31
	3	716	3260	2003
	4	2	6	60
	5	9	5	20
Average SSR size (bp)	1	470	–	–
	2	–	29	50.53
	3	82.01	48.52	41.36
	4	778	104.83	57.23
	5	396.78	121	81.1

## Data Availability

The *H. lacustris* raw sequencing reads have been deposited in the SRA (Sequence Read Archive) data resource of the NCBI with the Bioproject ID PRJNA904687 and PRJNA910497. The genome assembly files with their gene and repeat annotations are available at https://doi.org/10.5281/zenodo.11106870. For reviewing purposes, the genome and the annotation files can be downloaded at the following link: https://zenodo.org/records/11106870?token=eyJhbGciOiJIUzUxMiJ9.eyJpZCI6IjZhZGU0NTRiLTljMjEtNGE3OC05Yjg3LTQwZTRhNjZhNjZlYiIsImRhdGEiOnt9LCJyYW5kb20iOiIzM2JjODRhZjFkYjMwMzJhYmNjZDhkNTA1ZmJlZGY2ZiJ9.31VLnqaK_weVTuMOMz7-7xuimAyMcBd 8-2QJPYz_4vYD7XgxDzIkNFVfbZ3VBIpvfQ19_V62vm6TT3YLox7S3g.
