# Supplementary material for: Haematococcus lacustris genome assembly and annotation reveal diploid genetic traits and stress-induced gene expression patterns
Source: Algal Res. Author manuscript; Available in PMC 2024 Dec 23. (PMC7617258; doi:10.1016/j.algal.2024.103567)
Supplement: Supplemental Tables 1-8 and Figures 1-10 [file EMS201633-supplement-Supplemental_Tables_1_8_and_Figures_1_10.pdf]

***Haematococcus lacustris* genome assembly and annotation reveal diploid genetic traits and stress-induced gene expression patterns**

Luca Marcolungo[1‡], Francesco Bellamoli[1‡], Michela Cecchin[1‡], Giulia Lopatriello[1], Marzia Rossato[1], Emanuela Cosentino[1], Stephane Rombauts[2], Massimo Delledonne[1], Matteo Ballottari[1*]

[1] *Dipartimento di Biotecnologie, Università di Verona, Strada Le Grazie 15, 37134 Verona, Italy.*

[2]*Bioinformatics and Evolutionary Genomics, University of Ghent, Technologiepark 927, B-9052 Gent, Belgium*

*Address for correspondence: Matteo Ballottari, Dipartimento di Biotecnologie, Università di Verona, Strada le Grazie 15, 37134 Verona Italy; Tel: +390458027807; E-mail: matteo.ballottari@univr.it

‡These authors contributed equally.

SUPPLEMENTARY INFORMATION

Table 1: Summary of raw Oxford Nanopore Technologies (ONT), Illumina and PacBio (RSII) sequencing data

	ONT (MinION)	Illumina	PacBio RSII
Number of reads	2,061,059	119,607,416	2,828,818
Total bases (Gbp)	11.9	35.9	21.8
Reads N50	15,852	150 pair ends	11,507
Expected fold coverage	41X	239X	70X

Table 2: Statistics of ILLUMINA reads alignment. The raws show the average insert size, percentage of reads marked as duplicates, mapped coverage on the genome, percentage of the genome covered by at least 1, 2, 3, 5, 10, 20 and 30 reads, percentage of callable bases on the target for standard read depth (>3), FOLD 80 base penalty, the uniformity of coverage value, the total number of identified variants and the number of heterozygous variants.

**Number of input fragments**	119,607,416
**Mean insert size (bp)**	277.02
**Percentage of duplication**	14.71%
**Average coverage**	162.68
**%1X**	90.42
**%2X**	89.36
**%3X**	88.49
**%5X**	87.53
**%10X**	86.05
**%20X**	83.96
**%30X**	81.80
**PASS**	64.59%
**PASS DP10**	63.92%
**Fold80**	9.84
**Uniformity of coverage**	75.53
**Variants**	2,314,504
**Hetorizygous variants**	2,301,487

Table 3: Scaffolds length and Illumina average coverage. For the different scaffolds resulting from *H. lacustris* genome assembly the length and the average coverage of Illumina sequencing is reported.

Scaffold	Length (bp)	Average coverage (Illumina)
1	2,812,637	87.86
2	3,747,186	101.17
3	3,594,925	111.16
4	6,526,927	106.81
5	2,019,974	90.60
6	3,814,197	137.62
7	3,657,109	97.83
8	4,383,566	79.98
9	2,664,046	109.83
10	4,010,071	117.68
11	3,768,808	85.71
12	2,507,449	128.95
13	3,981,458	95.10
14	5,625,641	96.27
15	3,918,932	106.45
16	4,134,279	87.19
17	5,297,926	93.39
18	4,098,524	90.18
19	3,473,190	84.67
20	9,907,970	91.00
21	5,544,962	93.19
22	3,098,131	97.98
23	9,464,668	81.85
24	5,436,016	87.25
25	5,488,765	88.67
26	6,560,052	102.97
27	3,894,193	82.12
28	2,529,471	89.36
29	3,956,454	78.97
30	3,613,637	93.27
31	1,735,284	87.70
32	1,926,294	76.99

Table 4: Number of putative telomeric motifs identified at the ends of *H. lacustris* assembled genome scaffolds. AAGGATGGAC and AACCCT motifs are telomere sequence motifs for *Chlamydomonadales* order while TTTTAGGG is the telomeric motif found in *Chlamydomonas reinhardtii*.

Searched motifs:	AAGGATGGAC		AACCCT		TTTTAGGG	
	Number of identified motifs located at the first 50Kb of the scaffold	Number of identified motifs located at the latest 50Kb of the scaffold	Number of identified motifs located at the first 50Kb of the scaffold	Number of identified motifs located at the latest 50Kb of the scaffold	Number of identified motifs located at the first 50Kb of the scaffold	Number of identified motifs located at the latest 50Kb of the scaffold
H.lacustris_scaffold1	0	0	24	35	0	1
H.lacustris_scaffold2	0	0	50	19	0	0
H.lacustris_scaffold3	1	0	44	15	0	0
H.lacustris_scaffold4	0	0	27	13	0	1
H.lacustris_scaffold5	0	0	26	107	0	1
H.lacustris_scaffold6	0	0	33	27	1	0
H.lacustris_scaffold7	0	0	30	26	0	0
H.lacustris_scaffold8	0	0	18	20	0	0
H.lacustris_scaffold9	0	0	24	13	0	0
H.lacustris_scaffold10	0	0	37	27	0	0
H.lacustris_scaffold11	0	0	10	746	0	1
H.lacustris_scaffold12	0	0	19	31	0	0
H.lacustris_scaffold13	0	0	29	20	1	0
H.lacustris_scaffold14	0	0	43	54	0	3
H.lacustris_scaffold15	0	0	18	30	0	1
H.lacustris_scaffold16	0	0	24	41	1	2
H.lacustris_scaffold17	0	0	103	39	0	0
H.lacustris_scaffold18	0	0	41	22	0	1
H.lacustris_scaffold19	0	0	20	35	0	1
H.lacustris_scaffold20	0	0	31	22	1	2
H.lacustris_scaffold21	0	0	18	186	0	0
H.lacustris_scaffold22	0	0	26	16	0	0
H.lacustris_scaffold23	0	0	22	50	0	0
H.lacustris_scaffold24	0	0	10	15	1	0
H.lacustris_scaffold25	0	0	43	34	0	1
H.lacustris_scaffold26	0	0	37	22	0	0
H.lacustris_scaffold27	0	0	26	31	0	1
H.lacustris_scaffold28	0	0	41	17	2	0
H.lacustris_scaffold29	0	0	29	19	0	0
H.lacustris_scaffold30	0	0	36	23	2	0
H.lacustris_scaffold31	0	0	36	30	0	0
H.lacustris_scaffold32	0	0	24	15	0	0

Table 5: Pigment analysis of *Haematococcus lacustris* cells grown in different conditions. Chlorophyll to carotenoid ratio (Chl/Car) and chlorophyll a/b ratio (Chl a/b) were estimated from absorption spectra of pigment extracts as described in (Perozeni *et al.*, 2020). Total carotenoid content (Car tot) was set to 100, while total chlorophyll content (Chl tot) was calculated from Chl/Car ratio. Carotenoid content was analysed by HPLC (Perozeni *et al.*, 2020). Neo: neoxanthin; Viola: violaxanthin; Anthera: antheraxanthin; Lute: lutein; Zea: zeaxanthin; Cantha: canthaxanthin; Asta: astaxanthin; β-car: beta-carotene. Errors are repored as standard deviation (SD, n=3).

	**Neo**	**Viola**	**Anthera**	**Lute**	**Zea**	**Cantha**	**Asta**	**β-Car**	**Car Tot**	**Chl/Car**	**Chl a/b**	**Chl Tot**
**LL**	8,53	23,72	1,46	34,18	N.D	N.D	9,42	22,69	100,00	2,50	2,29	250,37
S.D.	*5,23*	*2,97*	*0,86*	*12,09*	--	--	*1,33*	*2,27*	*0,00*	*0,05*	*0,08*	*4,95*
**LL-N**	5,30	12,44	1,07	19,53	N.D	N.D	51,38	10,28	100,00	1,16	3,37	116,27
S.D.	*1,02*	*2,58*	*0,20*	*2,91*	--	--	*9,55*	*3,85*	*0,00*	*0,05*	*0,23*	*5,10*
**HL**	2,42	1,98	2,19	5,34	1,72	1,44	80,91	4,00	100,00	0,20	8,65	20,23
S.D.	*0,29*	*1,00*	*0,35*	*0,52*	*0,28*	*0,30*	*0,14*	*0,70*	*0,00*	*0,02*	*1,46*	*2,06*
**HL-N**	0,72	1,13	1,32	1,86	1,04	0,96	91,56	1,42	100,00	0,09	16,63	9,20
S.D.	*0,55*	*0,22*	*0,09*	*0,99*	*0,07*	*0,10*	*1,44*	*0,27*	*0,00*	*0,02*	*3,00*	*1,57*

Table 6: Identification of repeats in *Haematococcus lacustris* genome and comparison with the *Chlamydomonas reinhardtii* case.

	Chlamydomonas reinhardtii	***Haematococcus lacustris***
**Assembly length (bp)**	111,100,715	150,042,165
**Repetitive content (bp)**	24,697,036 (22.23%)	70,927,109 (47.27%)

Table 7. Codon usage in *Haematococcus lacustris* genome. The codon usage table gives for each codon: i. Sequence of the codon. ii. The encoded amino acid. iii. The proportion of usage of the codon among its redundant set, i.e. the set of codons which code for this codon's amino acid. iv. The expected number of codons, given the input sequence(s), per 1000 bases. v. The observed number of codons in the input sequences.

#Codon	AA	Fraction	Frequency	Number
GCA	A	0.228	29.408	190996
GCC	A	0.367	47.369	307645
GCG	A	0.209	27.000	175357
GCT	A	0.196	25.286	164228
TGC	C	0.773	12.858	83510
TGT	C	0.227	3.783	24568
GAC	D	0.750	33.795	219487
GAT	D	0.250	11.245	73034
GAA	E	0.152	8.206	53297
GAG	E	0.848	45.747	297113
TTC	F	0.664	17.106	111101
TTT	F	0.336	8.644	56142
GGA	G	0.092	7.637	49598
GGC	G	0.491	40.816	265089
GGG	G	0.291	24.160	156914
GGT	G	0.126	10.493	68147
CAC	H	0.712	17.409	113065
CAT	H	0.288	7.055	45821
ATA	I	0.102	2.714	17626
ATC	I	0.665	17.734	115179
ATT	I	0.234	6.238	40515
AAA	K	0.151	5.289	34351
AAG	K	0.849	29.769	193344
CTA	L	0.051	5.575	36206
CTC	L	0.136	14.796	96094
CTG	L	0.626	67.866	440770
CTT	L	0.059	6.357	41284
TTA	L	0.015	1.666	10817
TTG	L	0.112	12.149	78904
ATG	M	1.000	22.612	146857
AAC	N	0.783	17.738	115205
AAT	N	0.217	4.922	31965
CCA	P	0.212	13.542	87952
CCC	P	0.385	24.623	159920
CCG	P	0.174	11.110	72157
CCT	P	0.230	14.732	95678
CAA	Q	0.179	9.650	62677
CAG	Q	0.821	44.144	286699
AGA	R	0.047	2.871	18647
AGG	R	0.177	10.811	70216
CGA	R	0.074	4.491	29167
CGC	R	0.382	23.314	151418
CGG	R	0.223	13.592	88277
CGT	R	0.097	5.945	38613
AGC	S	0.411	29.903	194212
AGT	S	0.090	6.547	42522
TCA	S	0.127	9.227	59928
TCC	S	0.205	14.921	96910
TCG	S	0.082	5.937	38562
TCT	S	0.085	6.172	40087
ACA	T	0.205	10.293	66853
ACC	T	0.453	22.768	147870
ACG	T	0.186	9.372	60869
ACT	T	0.156	7.863	51069
GTA	V	0.056	3.890	25266
GTC	V	0.213	14.723	95622
GTG	V	0.654	45.089	292840
GTT	V	0.077	5.277	34273
TGG	W	1.000	14.174	92057
TAC	Y	0.767	14.896	96748
TAT	Y	0.233	4.529	29417
TAA	*	0.147	0.316	2052
TAG	*	0.280	0.602	3912
TGA	*	0.572	1.229	7983

Table 8. Comparison of *Haematococcus lacustris* chloroplast and mitochondrial genome with other known microalgae organelle genomes.

	Haematoccocus lacustris	***Chlorella vulgaris (Cecchin et al., 2019)***	***Chromochloris zofingiensis (Roth et al., 2017)***	***Chlamydomonas reinhardtii (Blaby et al., 2014)***	***Chlorella variabilis NC64A (Blanc et al., 2010)***
**Chloroplast genome**					
**Sequenced genome size**	1423 Kb	165 Kb	181 Kb	204 Kb	125 Kb
**Annotated protein coding genes**	75	71	71	68	79
**Annotated rRNAs**	9	4	6	10	3
**Annotated tRNAs**	84	46	31	29	31
**Mitochondrial genome**					
**Sequenced genome size**	145 Kb	92 Kb	42 Kb	16 Kb	78 Kb
**Annotated protein coding genes**	8	14	22	8	32
**Annotated rRNAs**	23	4	6	14	3
**Annotated tRNAs**	3	30	24	3	27

Figure 1. *Haematococcus lacustris* genome assembly pipeline

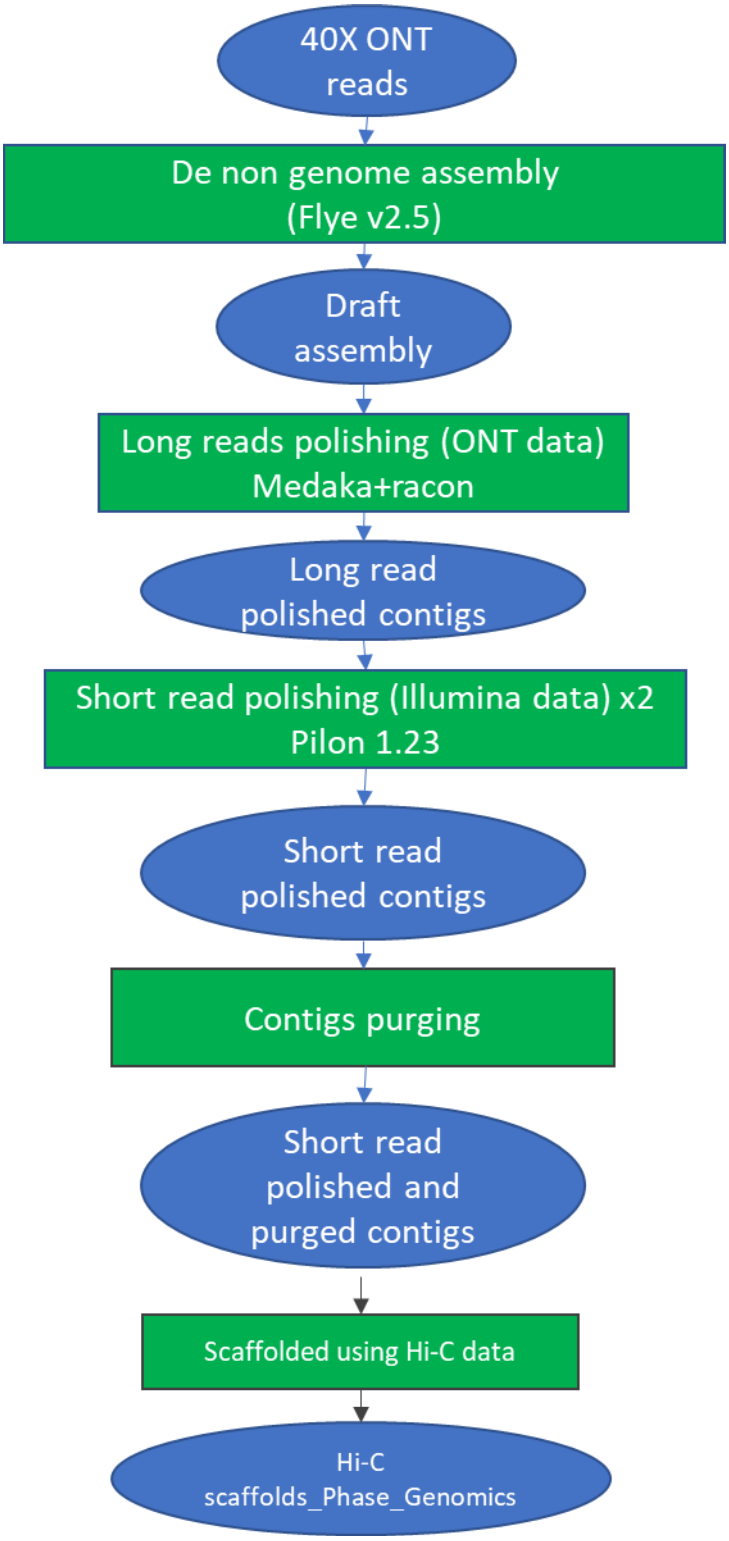

Figure 2: *Haematococcus lacustris* nuclear genome annotation workflow

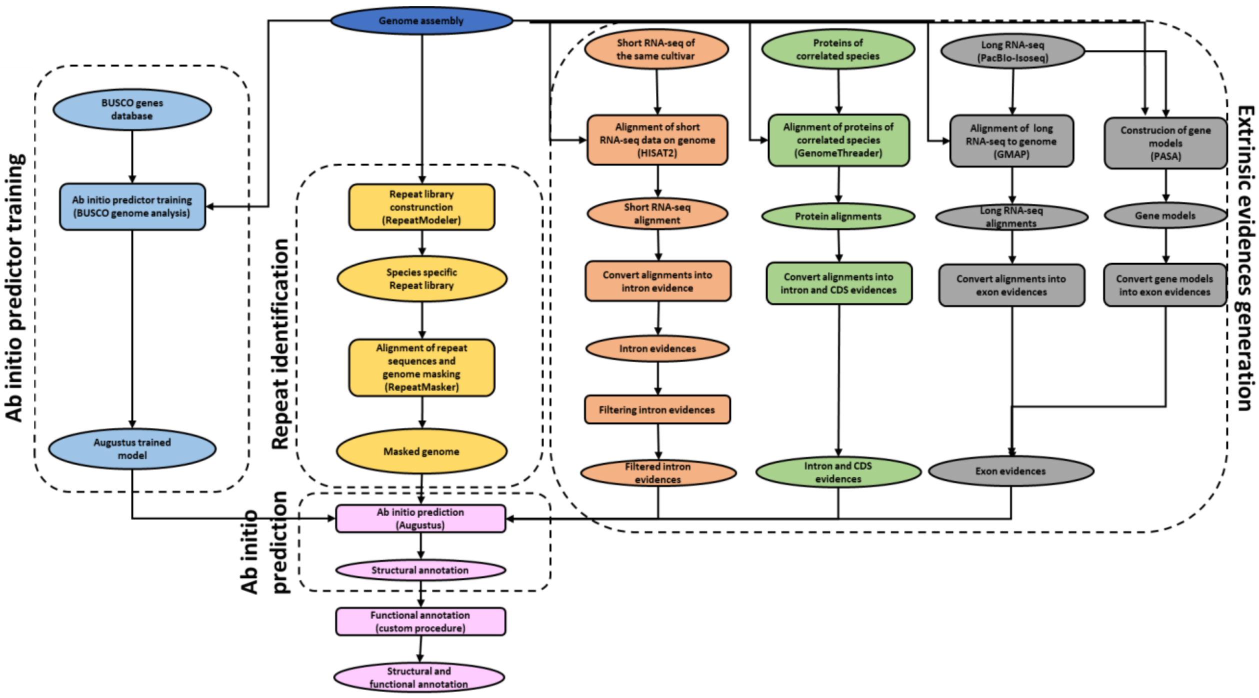

Figure 3. *Haematococcus lacustris* cells grown in low/high light in nitrogen replete medium or in nitrogen starvation. Transmission light microscopy images of *H. lacustris* cells grown for 3 days under different conditions: low light (40 µmol photons m^{-1} s^{-1}) with (LL) and without (LL-N) nitrogen and high light (400 µmol photons m^{-1} s^{-1}) with (HL) or without nitrogen (HL-N).

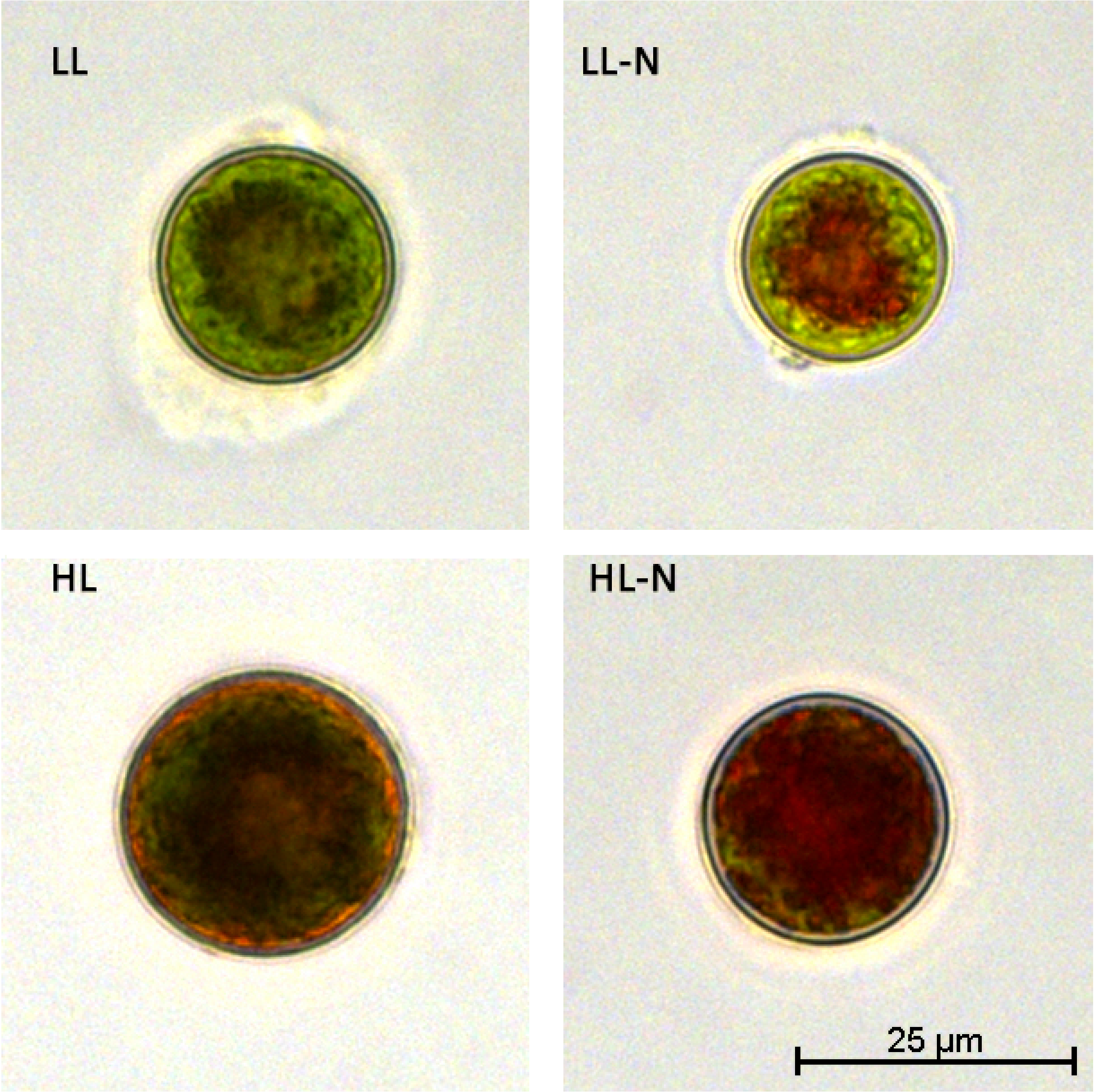

Figure 4: Integrative Genome Browser visualization of repetitive elements annotated in the *Haematococcus lacustris* genome assembly. (a) and (b) show two examples of short tandem repeats forming low-complexity regions of 25 and 34 kbp, respectively

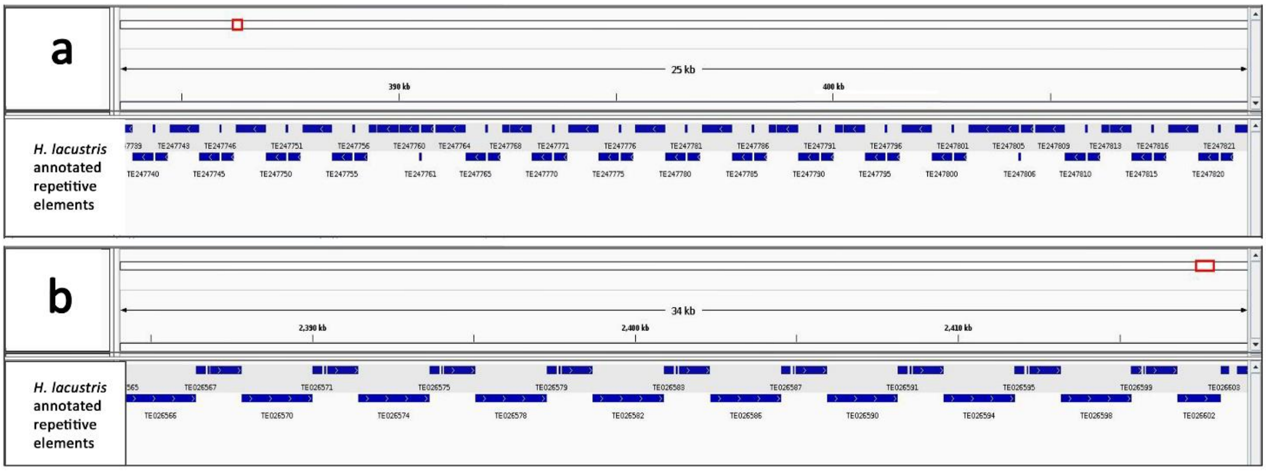

.

Figure 5. RNAseq samples distance matrix.

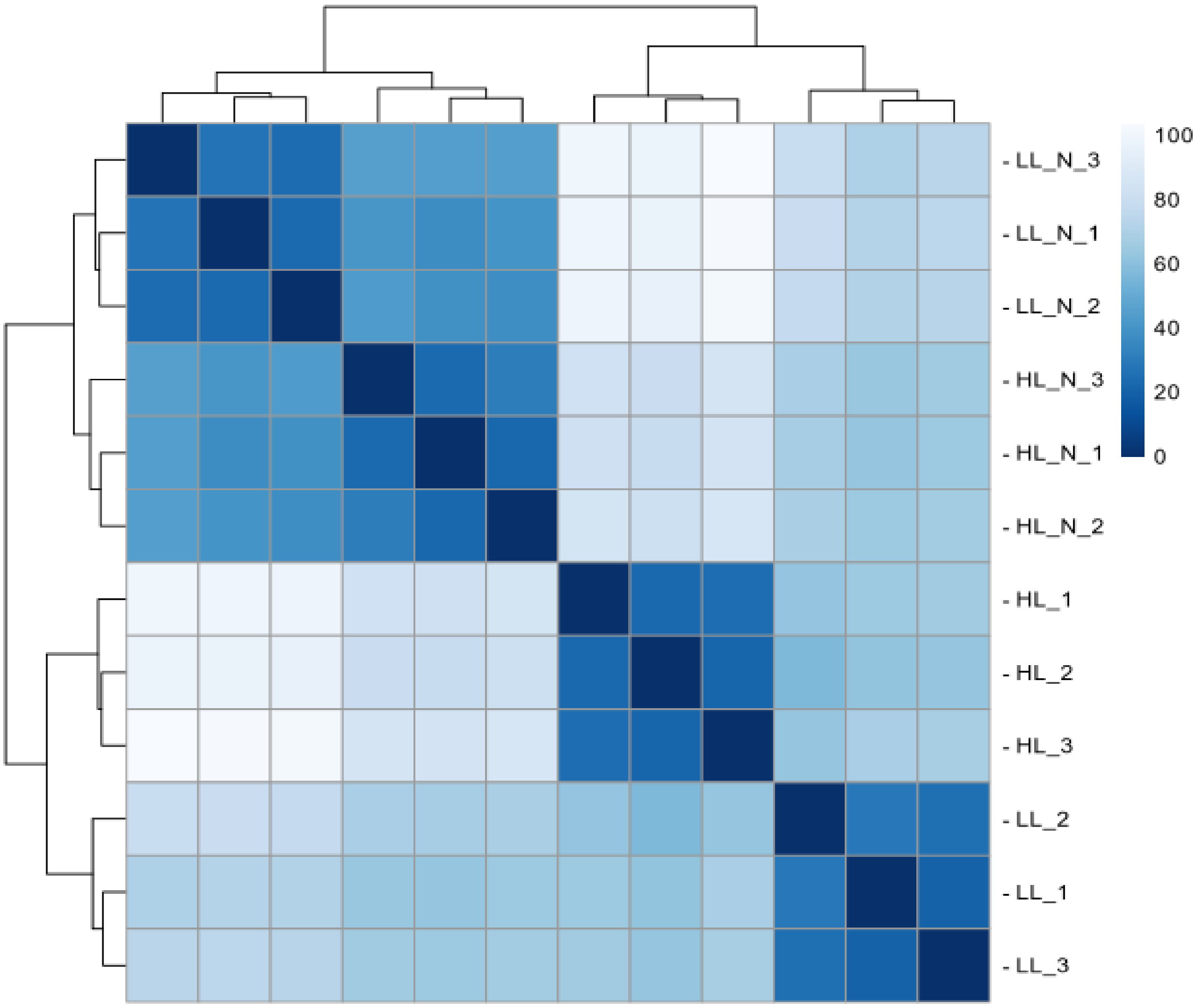

Figure 6. Comparison of psbs gene expression in Haematococcus lacustris cells exposed to differen growth conditions. Log2fold change of gene expression for g8651 gene, identified as the gene ecnodning for PSBS protein in H. lacustris, in cells grown in high light (HL), low light (LL), high light in nitrogen starvation (HL-N), or low light in nitrogen starvation (LL-N). Adjusted p-values are reported for each comparison.

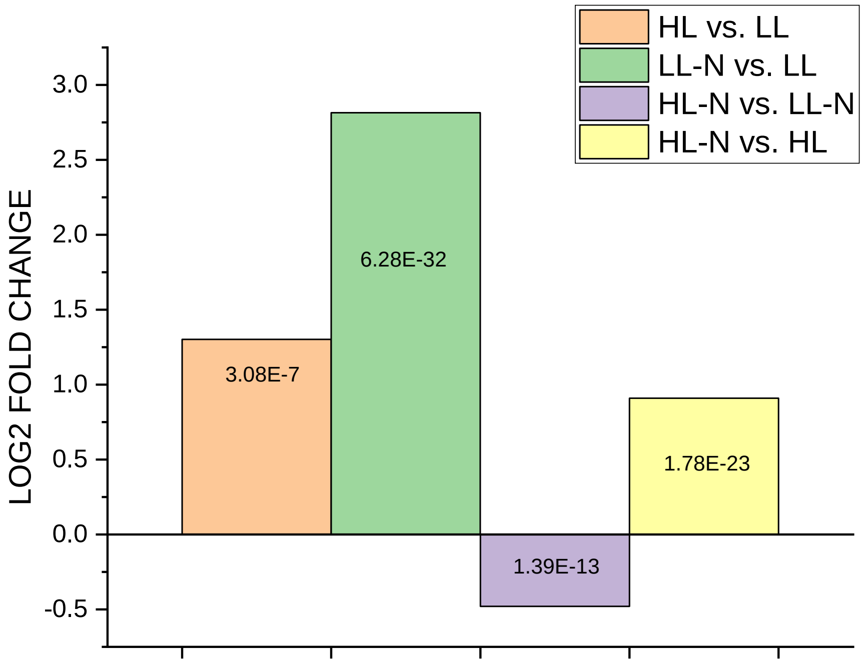

Figure 7. Terpenes biosynthetic pathway. Metabolic pathway diagram was retrieved from KEGG Mapper tool (Kanehisa and Goto, 2000, Kanehisa *et al.*, 2016, Kanehisa *et al.*, 2017). In red the genes upregulated in HL-N vs. HL.

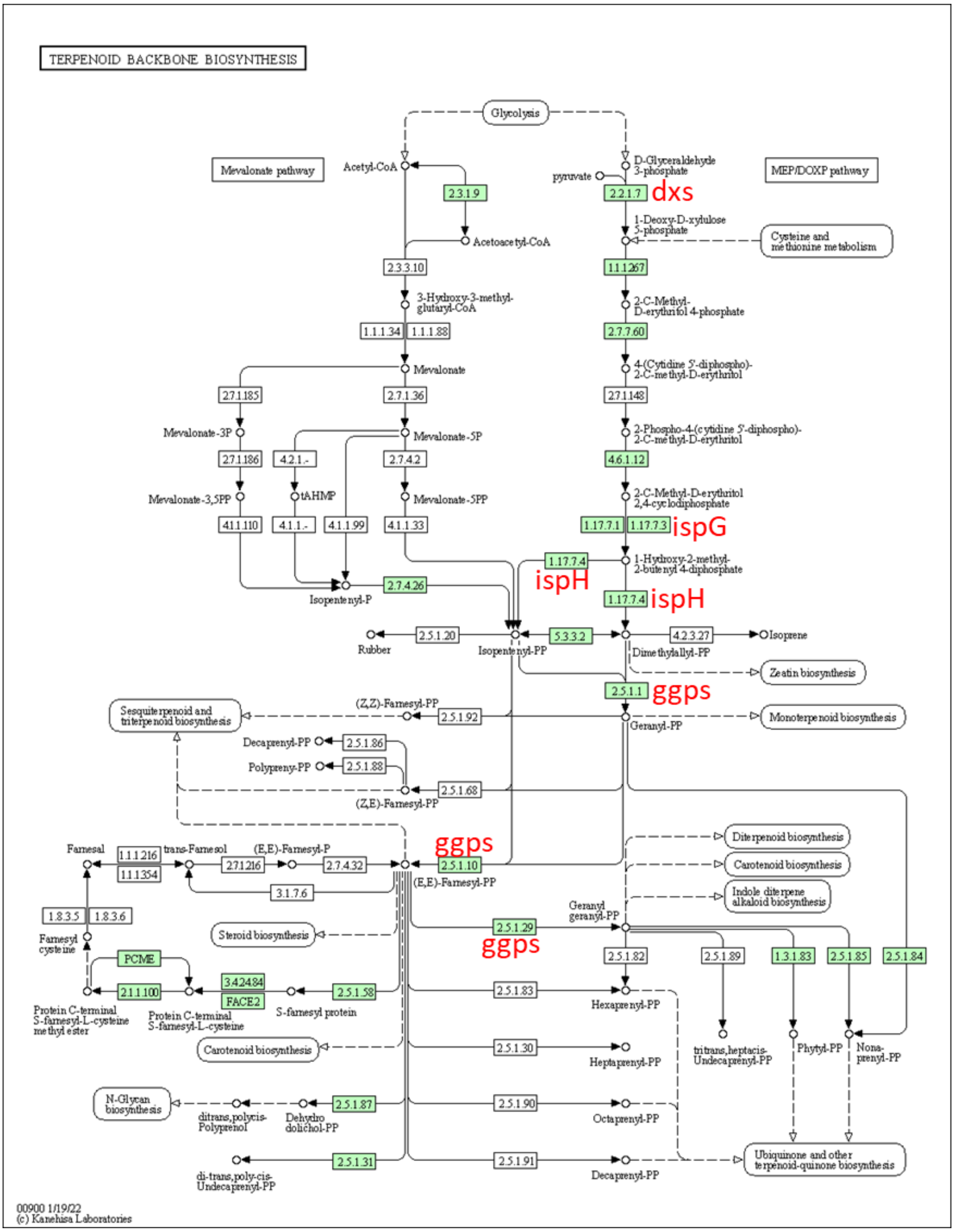

Figure 8. BKT enzymes encoded in *Haematococcus lacustris* genome. Protein alignment with BKT protein previously identified in *Haematococcus* (AANO3483.1)**.**

```
AAN03484.1      MPSESSDAARPVLKHAYKPPASDAKG-ITMALTIIGTWTAVFLHAIFQIRLPTSMDQLHW    59
g1780           MSSTSHN------IRFYDRDVSAALNIRRCAPQIIKSSGVLFLHAIFQIKLPTSLDQLHW    54
g8702           ------------------------------------------------------MDQLHW    6
                                                                      :*****

AAN03484.1      LPVSEATAQLLGGSSSLLHIAAVFIVLEFLYTGLFITTHDAMHGTIALRNRQLNDLLGNI    119
g1780           LPVSEATAQLVGGSSSLMHIAVVFFVLEFLYTGLFITTHDAMHGTIAMRNRQLNDFLGRV    114
g8702           LPVSEATAQLLGGSSSLLHIAAVFLVLEFLYTGLFITTHDAMHGTIALRNRQLNDLLGNI    66
                **********:******:***.**:**********************:*******:**.:

AAN03484.1      CISLYAWFDYSM----HWEHHNHTGEVGKDPDFHKGNPGLVPWFASFMSSYMSLWQFARL    175
g1780           CISLYAWFDYNMLHRKHWEHHNHTGEVGKDPDFHRGNPGIVPWFASFMSSYMSMWQFARL    174
g8702           CISLYAWFDYSMLHRKHWEHHNHTGEVGKDPDFHKGNPGLVPWFASFMSSYMSLWQFARL    126
                **********.*    ******************:****:*************:******

AAN03484.1      AWWAVVMQTLGAPMANLLVFMAAAPILSAFRLFYFGTYLPHKPEPGPAAGS--QVMSWFR    233
g1780           AWWTVVMQLLGAPMANLLVFMAAAPILSAFRLFYFGTYMPHKPEPSAASGSSPAVMNWWK    234
g8702           AWWAVVMQMLGAPMANLLVFMAAAPIFSAFRLFYYGTYLPHKPDPGPAAGS--QVMAWFR    184
                ***:**** *****************:*******:***:****:*. *:**   ** *::

AAN03484.1      AKTSEASDVMSFLTCYHFDL--------FAPWWQLPHCRRLSGRGLVPALA     276
g1780           SRTSQASDLVSFLTCYHFDLHWEHHRWPFAPWWELPNCRRLSGRGLVPA--     283
g8702           AKTSEASDVMSFLTCYHFDLHWEHHRWPFAPWWQLPHCRRLSGRGLVPALA     235
                ::**:***::**********        *****:**:************
```

Figure 9. Glycolysis, gluconeogenesis and TCA cycle. Metabolic pathway diagram was retrieved from KEGG Mapper tool (Kanehisa and Goto, 2000, Kanehisa *et al.*, 2016, Kanehisa *et al.*, 2017)**.** Red arrows represent genes upregulated in HL-N vs. HL, blue arrows are reported for genes upregulated in HL vs. LL, black arrow indicates genes downregulated in HL vs. LL.

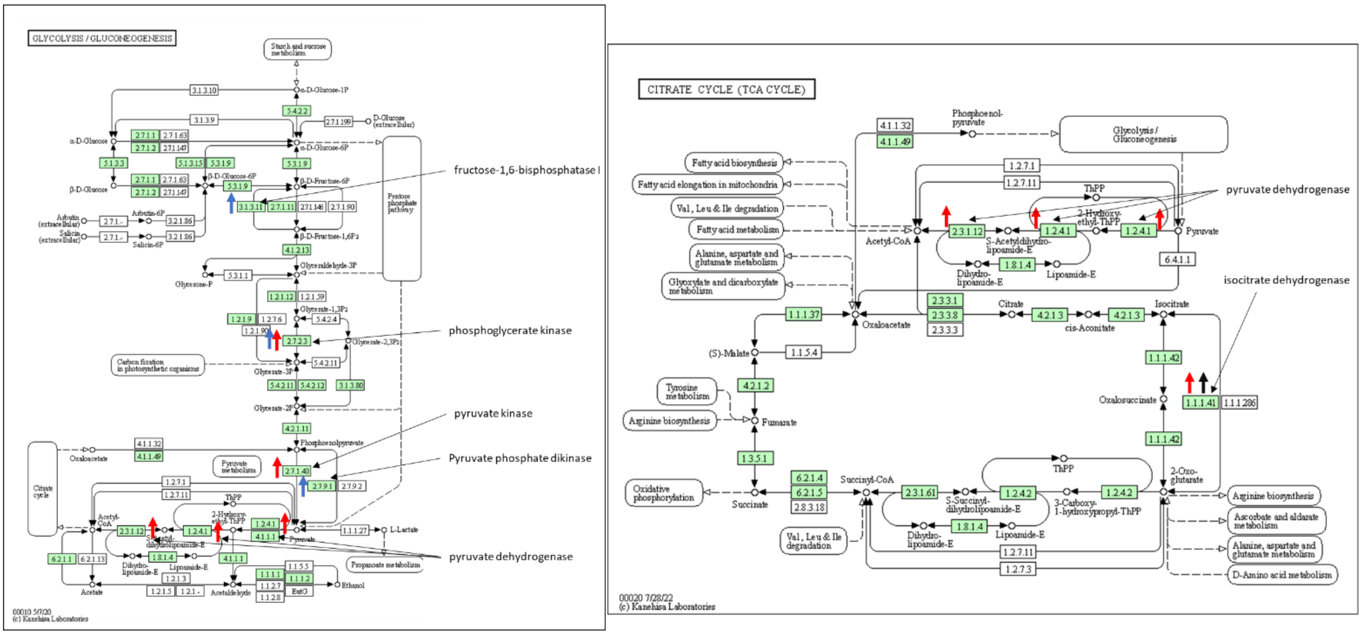

Figure 10. Alignment of MLDP proteins identified in *Haematococcus lacustris* with HOGP. HOGP is a lipid binding protein previously isolated from astaxanthin-rich cytoplasmic oil globules of *Haematococcus* (Peled et al., 2011).

```
              cov    pid    1 [        .         .         .         .         :         .         .         . 80
1 HOGP      100.0% 100.0%     MSEKQLKRLGFVHQGASYAYSYTGTAEKLYKTARSFAPTFVEPTLAQVEDRVVAITAPVVAQAQDLSEKALHIADDQVDC
2 g8.t1     100.0%  85.5%     MSETPLRRLGFVQQGASYAYSYTGTAEKLYKTVRSFAPTFVEPHLSNLEDKAVAITAPVVAQVQDLSEKALHIADDQVDC
3 g2545.t1 100.0%  99.6%     MSEKQLKRLGFVHQGASYAYSYTGTAEKLYKTARSFAPTFVEPTLAQVEDRVVAITAPVVAQAQDLSEKALHIADDQVDC

              cov    pid   81          .         1         .         .         .         .         :         . 160
1 HOGP      100.0% 100.0%     ILNTTDKAVADGKKGVVDCMNGVKEMHEKNMQTYIATSNSYFEYIKGISDWAKDKLNPIKGGQHALDTLNAAIAKAQEAT
2 g8.t1     100.0%  85.5%     LLSTADKAVAGSKKGVADSLTGVKKMHEKNMQAYQAASNSYFEYIKGISDWAKDKLNPIKGGQLALDTLNAAIAKAQEAT
3 g2545.t1 100.0%  99.6%     ILNTTDKAVADGKKGVVDCMNGVKEMHEKNMQTYIATSNSYFEYIKGISDWAKDKLNPIKGGQHALDTLNAAIAKAQEAT

              cov    pid  161          .         .         .         2         .         .         .         . 240
1 HOGP      100.0% 100.0%     DPDVAAKMALDAWNSFASVPVVAKVLETADPVTQTGLSSFYKLHDTLVSWPLYSKVVSTGVSTLSWATTTMPYKLGAQYM
2 g8.t1     100.0%  85.5%     DPDVAAKMGMDAWSQFASVPVVAKVLETADPVTQSGLSSFYKLHDTLVSWPLYSKVVATGASTLSWAATTTPYKLGAQYM
3 g2545.t1 100.0%  99.6%     DPDVAAKMALDAWNSFASVPVVAKVLETADPVTQTGLSSFYKLHDTLVSWPLYSKVVSTGVSTLSWATTTTPYKLGAQYM

              cov    pid  241          :         .         .    ] 275
1 HOGP      100.0% 100.0%     YPLVQPVADPALAKITNSKVINGTLSYWKPTASAA
2 g8.t1     100.0%  85.5%     YPLVKPVADPALAKLTNSKVIADTLDYWKPTASAA
3 g2545.t1 100.0%  99.6%     YPLVQPVADPALAKITNSKVINGTLSYWKPTASAA
```

Blaby, I.K., Blaby-Haas, C.E., Tourasse, N., Hom, E.F., Lopez, D., Aksoy, M., Grossman, A., Umen, J., Dutcher, S., Porter, M., King, S., Witman, G.B., Stanke, M., Harris, E.H., Goodstein, D., Grimwood, J., Schmutz, J., Vallon, O., Merchant, S.S. and Prochnik, S. (2014) The Chlamydomonas genome project: a decade on. *Trends Plant Sci*, **19**, 672-680.

Blanc, G., Duncan, G., Agarkova, I., Borodovsky, M., Gurnon, J., Kuo, A., Lindquist, E., Lucas, S., Pangilinan, J., Polle, J., Salamov, A., Terry, A., Yamada, T., Dunigan, D.D., Grigoriev, I.V., Claverie, J.M. and Van Etten, J.L. (2010) The Chlorella variabilis NC64A genome reveals adaptation to photosymbiosis, coevolution with viruses, and cryptic sex. *Plant Cell*, **22**, 2943-2955.

Cecchin, M., Marcolungo, L., Rossato, M., Girolomoni, L., Cosentino, E., Cuine, S., Li-Beisson, Y., Delledonne, M. and Ballottari, M. (2019) Chlorella vulgaris genome assembly and annotation reveals the molecular basis for metabolic acclimation to high light conditions. *Plant Journal*, **100**, 1289-1305.

Kanehisa, M., Furumichi, M., Tanabe, M., Sato, Y. and Morishima, K. (2017) KEGG: new perspectives on genomes, pathways, diseases and drugs. *Nucleic Acids Res*, **45**, D353-D361.

Kanehisa, M. and Goto, S. (2000) KEGG: kyoto encyclopedia of genes and genomes. *Nucleic Acids Res*, **28**, 27-30.

Kanehisa, M., Sato, Y., Kawashima, M., Furumichi, M. and Tanabe, M. (2016) KEGG as a reference resource for gene and protein annotation. *Nucleic Acids Res*, **44**, D457-462.

Peled, E., Leu, S., Zarka, A., Weiss, M., Pick, U., Khozin-Goldberg, I. and Boussiba, S. (2011) Isolation of a novel oil globule protein from the green alga Haematococcus pluvialis (Chlorophyceae). *Lipids*, **46**, 851-861.

Roth, M.S., Cokus, S.J., Gallaher, S.D., Walter, A., Lopez, D., Erickson, E., Endelman, B., Westcott, D., Larabell, C.A., Merchant, S.S., Pellegrini, M. and Niyogi, K.K. (2017) Chromosome-level genome assembly and transcriptome of the green alga Chromochloris zofingiensis illuminates astaxanthin production. *Proc Natl Acad Sci U S A*, **114**, E4296-E4305.
